# Direct Electrical Access to the Spin Manifolds of Individual Lanthanide Atoms

**DOI:** 10.1021/acsnano.4c14327

**Published:** 2025-01-14

**Authors:** Gregory Czap, Kyungju Noh, Jairo Velasco, Roger M. Macfarlane, Harald Brune, Christopher P. Lutz

**Affiliations:** † IBM Almaden Research Center, 650 Harry Road, San Jose, California 95120, United States; ‡ Center for Quantum Nanoscience (QNS), Institute of Basic Science (IBS), Seoul 03760, Republic of Korea; § Department of Physics, Ewha Womans University, Seoul 03760, Republic of Korea; ∥ Department of Physics, University of California, Santa Cruz California 95064, United States; ⊥ Institute of Physics, École Polytechnique Fédérale de Lausanne (EPFL), Lausanne CH-1015, Switzerland

**Keywords:** quantum magnetism, STM, ESR, IETS, monovalent lanthanides, lanthanide magnetism, qudit

## Abstract

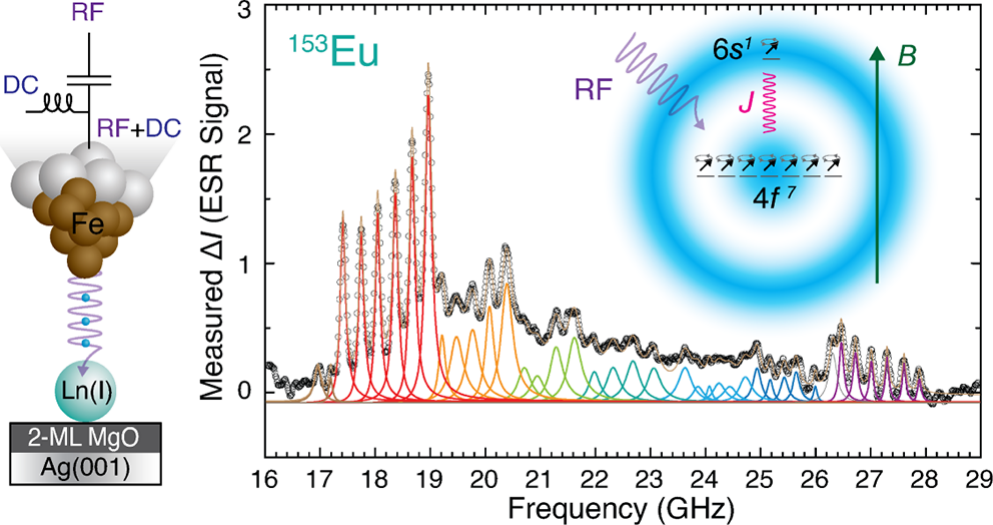

Lanthanide atoms show long magnetic lifetimes because of their strongly localized 4*f* electrons, but electrical control of their spins has been difficult because of their closed valence shell configurations. We achieved electron spin resonance of individual lanthanide atoms using a scanning tunneling microscope to probe the atoms bound to a protective insulating film. The atoms on this surface formed a singly charged cation state having an unpaired 6*s* electron, enabling tunnel current to access their 4*f* electrons. Europium spectra display a rich array of transitions among the 54 combined electron and nuclear spin states. In contrast, samarium’s ground state is a Kramers doublet with a very large *g*-factor of 5. These results demonstrate that all-electronic sensing and control of individual lanthanide spins is possible for quantum devices and spin-based electronics by using their rarely observed monovalent cation state.

## Introduction

Lanthanide atoms combine localized 4*f* electron spins with strong spin–orbit coupling, making them highly promising for single-ion magnetic memories and quantum bits. They exhibit magnetic bistability in single-ion molecular and atomic magnets,^1−3^ and very long coherence times in molecules^4−6^ or when dilutely dissolved in ionic crystals.^7−10^ Single lanthanide ions also offer efficient light–matter–spin interactions for use in quantum communication.^9,11^ Electron spin resonance (ESR) is a versatile technique capable of coherently controlling and addressing the properties of quantum spin systems, including access to hyperfine interactions between electron and nuclear spins.^12^ Combining ESR with scanning tunneling microscopy (STM) allows all-electronic spin manipulation of individual atoms, molecules, and assembled nanostructures in an environment controlled on the atomic scale.^13^ Thus far, spins in 3*d* adatoms^13−20^ and in spin-1/2 molecules^21−23^ have been addressed in ESR-STM, and spin resonance of lanthanide spins has been accessed indirectly through the use of a neighboring spin-resonant 3*d* adatom.^24^

Here we demonstrate that the open valence shell character of monovalent lanthanide atoms allows direct electrical driving, sensing, and control of their 4*f* atomic spins in an STM. We refer to lanthanides as monovalent when they retain their full set of free-atom *f* electrons (which usually results in divalent species in compounds) and when their cation state also leaves their valence electrons in an open-shell configuration such as 6*s*^1^. Monovalent lanthanides Eu(I) and Sm(I) have been proposed to exist as charged dopants in irradiated^25,26^ or additively reduced^26^ alkali metal halide crystals. Other monovalent lanthanides were observed only recently, in borozene complexes^27^ and in lanthanides on metal-supported graphene.^28^ The monovalent electron configuration is otherwise very uncommon for lanthanides.^27,29^ We obtained the monovalent state by choosing elements that occur readily as divalent species in compounds where they retain their full complement of 4*f* electrons, which include Eu and Sm.^30^ We find that Sm and Eu cations form a monovalent state when adsorbed on a thin film of insulating MgO grown on an Ag(100) surface, where they spontaneously ionize to the +1 charge state having the open 6*s*^1^ valence configuration.

## Results and Discussion

We present exceptionally rich ESR spectra of individual Eu^+^ and Sm^+^ ions. These ESR spectra are complemented by inelastic electron tunneling spectra (IETS) to probe the higher-energy magnetic excitations that allow determination of the electronic configurations. The *f*-shell occupations are half-full (4*f*^7^ for Eu) or nearly half-full (4*f*^6^ for Sm) which results in high *f-*spin configurations for both elements, but with markedly different properties. Eu has a large total spin *S* = 4 and vanishing orbital angular momentum (level ^9^S_4_). This enables access to transitions between crystal-field-split states using ESR-STM. The ESR spectra of Eu show at least four trees of peaks, each tree consisting of a hyperfine sextet, because of its large manifold of electronic and nuclear spin states and its unusually small magneto-crystalline anisotropy. In contrast, Sm has a Kramers doublet ground state (^8^*F*_1/2_) with nearly canceling spin and orbital angular momentum, resulting in an unusually large and anisotropic *g*-factor close to 5. For each odd-nucleon isotope of both elements, the ESR spectra reveal the hyperfine interaction *A*, electron *g*-factor, and for Eu the magneto-crystalline anisotropy parameters. These results show that individual lanthanide atomic spins with an open-shell valence configuration can be directly sensed and controlled electrically.

**Figure 1:**
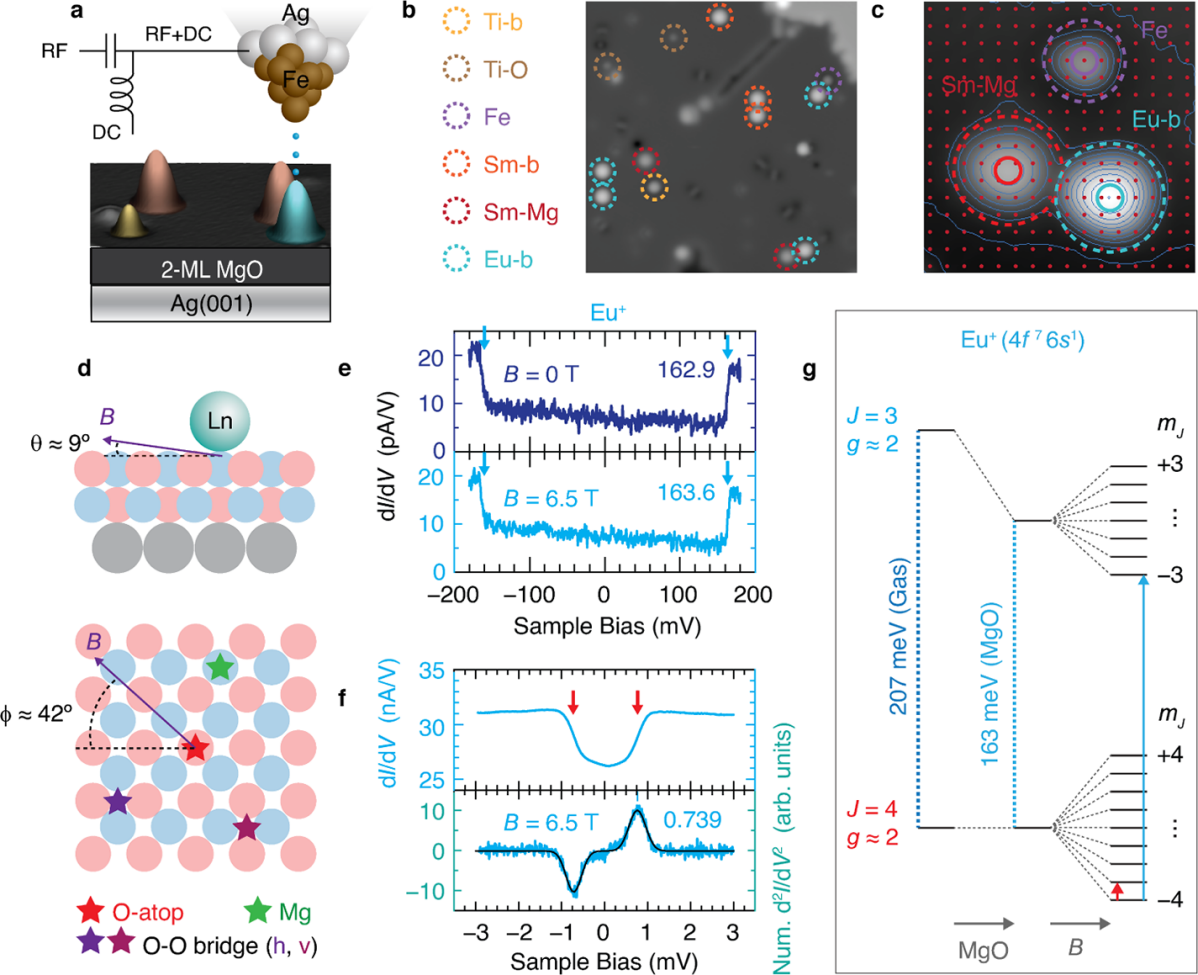
Lanthanide atoms on an MgO film and Eu excitations. (a) Schematic of the STM tip and sample consisting of adatoms on a two-monolayer epitaxial MgO film on Ag(001). (b) Constant-current STM image of adatoms as labeled (tip-height set point *I*_set_ = 20 pA, *V*_set_ = 60 mV, 24 nm square). Atoms were identified by their spectral features and their adsorption sites. (c) STM image used to determine binding sites (red points denote O atoms), set point 20 pA at 60 mV, 4.6 nm square. (d) Side and top views of MgO structure (red: O; blue: Mg; gray: Ag) showing lanthanide binding sites (star symbols as labeled) and applied magnetic field direction. (e,f) Inelastic tunneling spectra (d*I/*d*V*) of bridge-site Eu adatoms (Eu-b). (e) Excitations at ∼163 mV at 0 and 6.5 T with the tip positioned 0.1 nm farther away from the surface from tip height *I*_set_ = 4 pA at *V*_set_ = 80 mV. *V*_mod_ = 3 mV rms (root-mean-square) at *f* = 803 Hz. (f) Low-energy excitation appearing in a magnetic field *B* = 6.5 T, set point 300 pA at 10 mV, *V*_mod_ = 0.1 mV rms. The lower curve is d^2^*I*/d*V*^2^, numerically computed from the upper curve. (g) Schematic energy level diagram of gas-phase and MgO-adsorbed Eu^+^ ions. Application of a magnetic field *B* (right) splits the states (not shown to scale). Vertical arrows show the two types of observed inelastic excitations: *m*_*J*_-changing (red) and 6*s*-flip (blue).

Atoms of both elements were found to bind at three different sites on MgO: at the oxygen site (where they appear ∼0.3 nm tall), oxygen–oxygen bridge site (∼0.3 nm) and magnesium site (∼0.2 nm) (Figures 1b,c and S1). In contrast to other lanthanide elements on MgO thin films,^31−33^ we find that Eu and Sm have a preference to adsorb on bridge and Mg sites. We were able to readily reposition them among the bridge and Mg sites, and for some tips, the oxygen site, using atomic manipulation (see Methods).

### Europium Spin Excitations

When Eu was adsorbed on the bridge site (designated Eu-b) it exhibited IETS excitations seen as steps in the differential conductance (d*I/*d*V*) at 163 mV (Figure 1e). This excitation shifted with magnetic field (Figure 1e) and showed sensitivity to the spin polarization of the tip^34^ (Figure S2e), indicating that it is a magnetic excitation of the atom. Comparison with optical spectroscopy data for gas phase Eu ions,^35^ and further measurements below, indicate that the configuration of Eu-b is the one-fold positively charged ion Eu^+^ with a ground-state configuration of 4*f*^7^5*d*^0^6*s*^1^, which has an open valence shell as desired. Its electronic state has *f*-shell spin *S*_4*f*_ = 7/2, orbital angular momentum *L*_4*f*_ = 0, total *f*-shell angular momentum *J*_4*f*_ = 7/2, valence (*s*-shell) electron spin *S*_val_ = 1*/*2, total spin *S*_tot_ = 4, and total angular momentum *J*_tot_ = *S*_tot_ = 4. The 4*f* spin is aligned with the valence spin, giving the electronic level ^9^S_4_. The lowest excitation of gas-phase Eu^+^ makes a transition to ^7^S_3_, which corresponds to an intra-atomic spin flip of the 6*s* valence electron with respect to the 4*f* spin, which changes *J*_tot_ from 4 to 3. Its gas-phase excitation energy of 207 mV^35^ is comparable to our observed 163 mV conductance step (Figure 1e,g), to which we assign this “6*s*-flip” excitation. The smaller excitation energy for these surface-adsorbed atoms compared to gas-phase ions is likely due to partial charge transfer out of the 6*s* orbital.^28^ At high magnetic field, a gap in d*I/*d*V* opens close to zero bias voltage (Figure 1f) whose position gives the Zeeman energy. For an applied magnetic field *B* = 6.5 T this spin-flip excitation has an energy of 0.739 mV, yielding a *g*-factor *g* = 1.97 ± 0.02 for both bridge-site orientations of Eu-b (Figure S2), in agreement with *g* = 1.984 reported for the lowest gas-phase Eu^+^ multiplet.^35^ Eu-b is thus a high-spin cation with vanishing orbital moment. Its spin *S* = 4 is larger than the largest previously observed ground-state spin (*S* = 7/2 for trivalent Gd) for any atom or ion of any element in a solid-state environment.^36^

**Figure 2:**
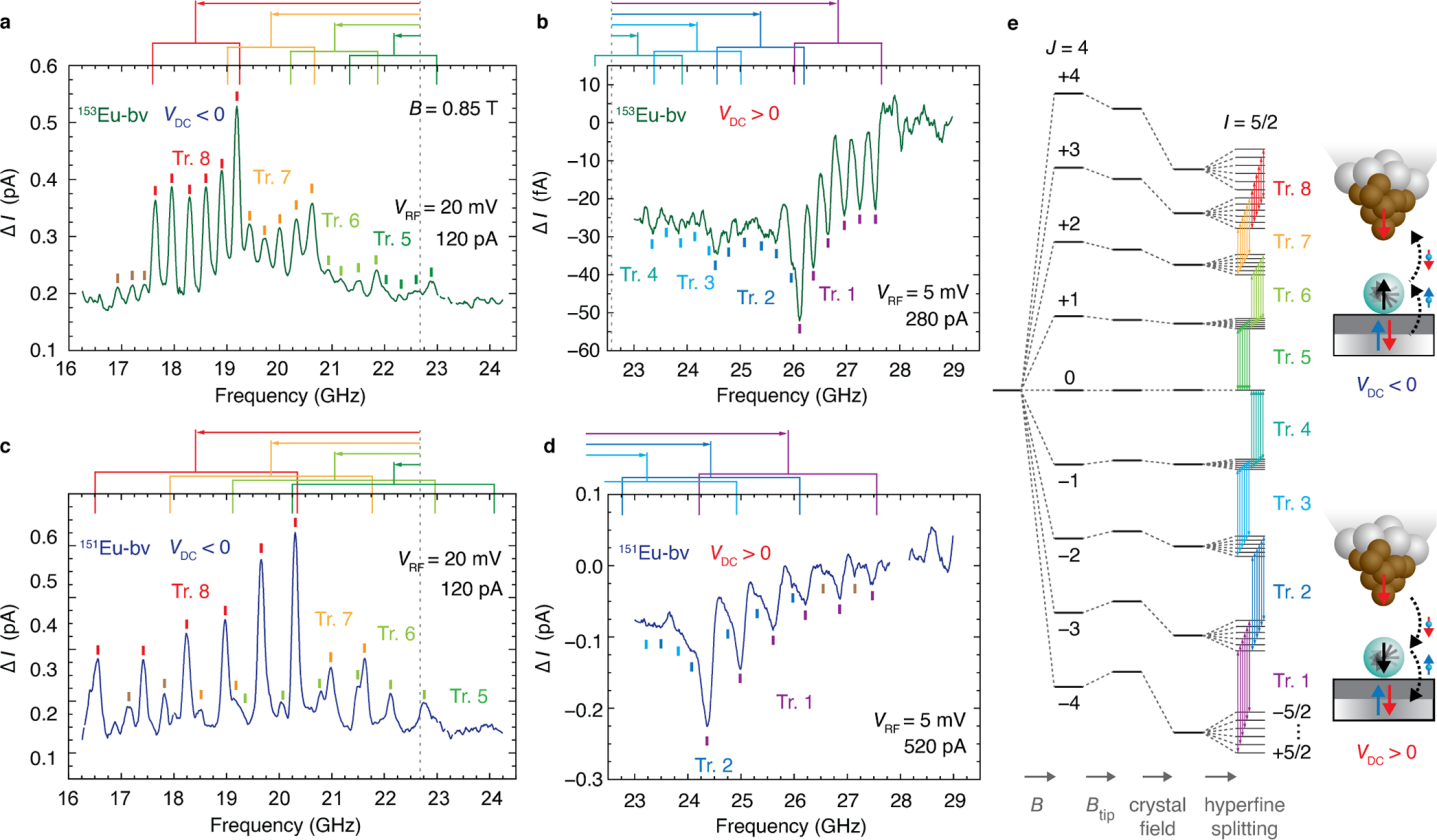
ESR spectra of Eu isotopes on MgO bridge sites and magnetic level scheme. (a, b) ^153^Eu-b (nuclear spin *I* = 5*/*2, natural abundance 52.19%) at negative (a) and positive (b) bias voltage *V*_DC_. Tic marks indicate peaks assigned to transitions shown in (e) with color code indicating tree number (initial-state *m*_*J*_); brown tics indicate peaks that may be weakly allowed transitions that change *m*_*I*_. Arrows at top show shift of each tree that is due mainly to the out-of-plane magneto-crystalline anisotropy *D*. (c, d) ^151^Eu-b (*I* = 5*/*2, 47.81%) at negative and positive bias. All spectra: set point *V*_set_ = 50 mV, *I*_set_ and radio frequency voltage *V*_RF_ (zero-to-peak) as shown in each panel, *T* = 1.2 K, *B* = 0.85 T; bias *V*_DC_ = −50 mV for (a) and (c), *V*_DC_ = +50 mV for (b) and (d). Data shown in (a–d) have been smoothed for clarity and certain data points are omitted in frequency intervals that are dominated by RF transmission artifacts; see Figure S3 for complete raw data sets. (e) Schematic magnetic energy levels of the ground-state multiplet, split by Zeeman energy due to *B* and *B*_tip_, perturbed by *D >* 0 and hyperfine splitting with *A >* 0 (shifts are exaggerated), to yield eight trees of transitions (colored lines) labeled Tr. 1 through Tr. 8. The rightmost schematics depict the electron spin-torque process at positive bias (bottom) and negative bias (top).

**Figure 3:**
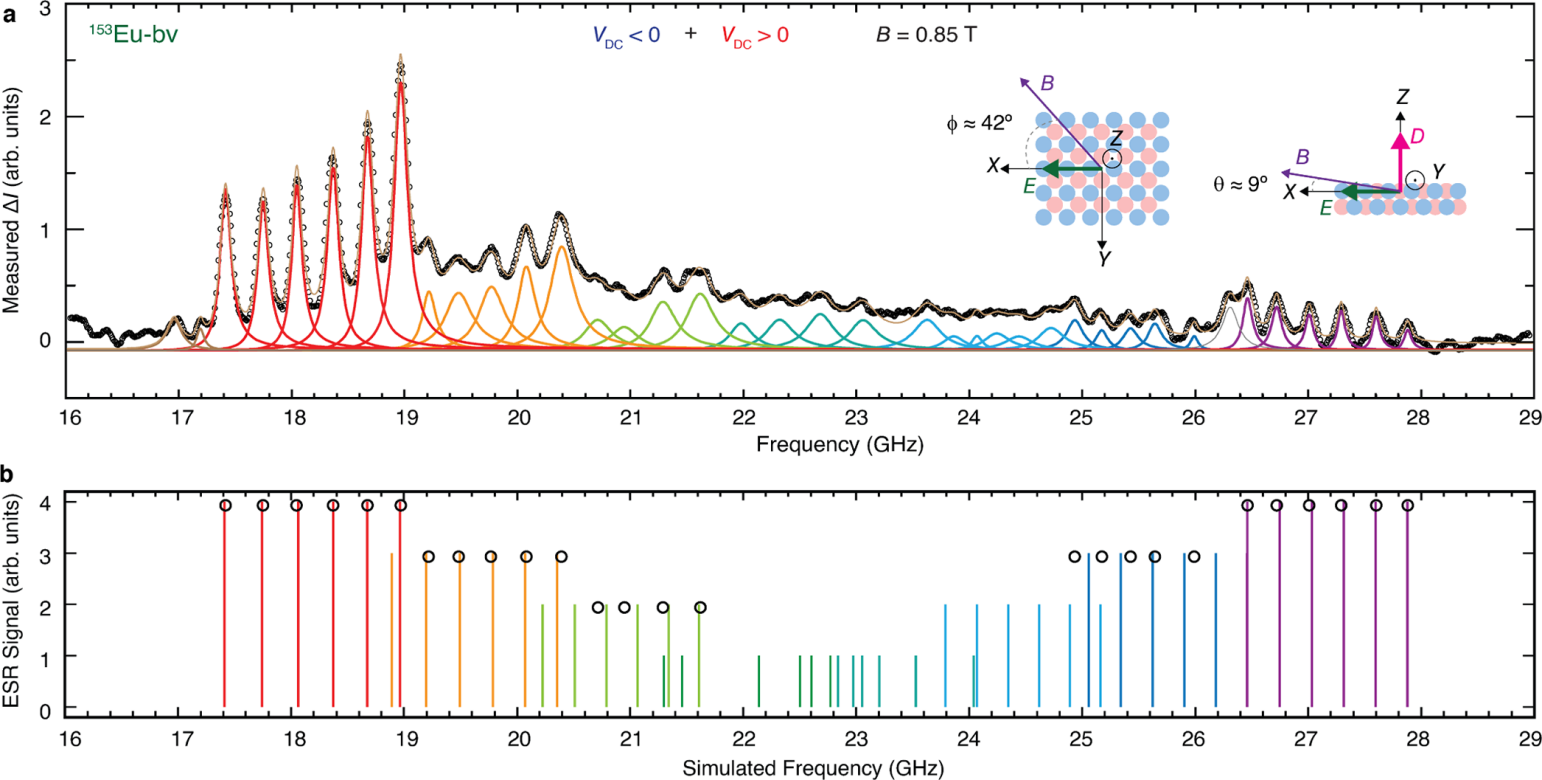
Broad-band ESR spectrum of Eu and Hamiltonian model transition energies. (a) ESR spectrum of bridge-site ^153^Eu over a wide frequency range that covers all eight hyperfine-split trees. Spectrum (black points) is the weighted sum of two smoothed spectra, one at each bias polarity (see Figure S6 for details), which renders the transitions at each extreme of the spin-flip energy range visible. Curves are Lorentzian peaks fit to the measured spectra using the color scheme of Figure 2e, and their sum (light brown). Brown peaks may be weakly allowed transitions; gray peak occurs at known artifact in the instrument transfer function. (b) Frequencies of allowed transitions determined from the model Hamiltonian with fit parameters *B*_tot_ = *B* + *B*_tip_ = 0.8175 T at angle 84° from the surface normal and 40° from MgO bridge-site O–O direction, *A* = 291 MHz, crystal field parameters *D* = 1515 MHz and *E* = 360 MHz along axes shown in (a) inset. Each transition is shown as a vertical line having height and color that indicates the dominant *m*_*J*_ component of the initial state but are otherwise arbitrary. Black circles represent fitted peak positions from the data in (a), with fits for trees 3–5 omitted due to large uncertainties in their peak positions. The spacing of peaks in trees 4 and 5 are not uniform because the initial or final state has *m*_*J*_*=* 0, for which the electron Zeeman energy vanishes, so other terms determine the properties.

Using the *g*-factor for Eu determined by the tunneling spectra, we investigate ESR by using a magnetic field where spin-flip transitions are positioned in the highest accessible frequency range for our microscope, ∼ 15–30 GHz. The ESR spectra of individual ^153^Eu-b and ^151^Eu-b atoms show an extremely rich peak structure (Figure 2), consisting of several trees of resonant peaks. Each tree consists of six hyperfine peaks that correspond to the six projections *m*_*I*_ of the nuclear spin *I* = 5*/*2 that is present in both isotopes. All previous ESR-STM spectra showing hyperfine peaks revealed only one such hyperfine-split tree,^14,19,37^ (Figure S4). Here we detect at least four such trees for the following reasons: First, the large electron total angular momentum *J*_tot_ = 4 admits many electron spin transitions among its principal-axis projections −4 ≤ *m*_*J*_ ≤ 4. Second, the axial magneto-crystalline anisotropy *D* is very small because of the absence of orbital angular momentum (*L* = 0 as a consequence of the exactly half-filled *f*-shell), which places all the spin-flip transitions close in energy. Third, by choosing the polarity of the bias voltage *V*_DC_, we are able to use spin torque^38^ to selectively populate either the more positive or more negative values of *m*_*J*_ as the initial state of ESR transitions (depicted schematically in Figure 2e). A weighted sum of spectra taken with opposite bias voltages gives a spectrum spanning the full range from trees 1 through 8 (Figure 3).

We find an excellent fit to the measured spectra for both isotopes and at both bias polarities by using the Hamiltonian
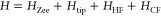
1where the electron Zeeman energy is *H*_Zee_ = *gμ*_*B*_**S·B**; the magnetic interaction between tip and sample spins is *H*_tip_ = *g***S·B**_tip_ where **B**_tip_ is the effective tip magnetic field; the dominant hyperfine (HF) interaction is *H*_HF_ = *A***S·I**; and the crystal field (CF) terms are
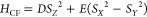
2

Here *X*, *Y*, and *Z* are the high-symmetry axes of the bridge binding site (Figure 3a inset). We omit the weaker nuclear quadrupole interaction and higher-order CF terms.

The Eu^+^ level scheme resulting from the Hamiltonian (Figure 2e) shows the Zeeman splitting of the nine *m*_*J*_ states by *B* and *B*_tip_. These nine electron spin states admit eight transitions compatible with selection rules that we take to require Δ*m*_*J*_ = ±1 and Δ*m*_*I*_ = 0. We label these transitions as tree 1 (*m*_*J*_ = −4 ↔ −3) through tree 8 (*m*_*J*_ = +3 ↔ +4). Each *m*_*J*_ state is split by the hyperfine interaction into six nuclear states *m*_*I*_, which results in six ESR peaks in each tree. Because the Zeeman energy is much larger than both the hyperfine splitting and crystal field splitting, the quantum states are well approximated as product states of electronic and nuclear spin eigenstates, and we label them by their dominant *m*_*J*_ and *m*_*I*_ components. Considering only the HF and Zeeman effects, all eight electron spin transitions (trees) would have similar excitation energies. The CF term lifts this degeneracy by generating a blueshift for trees 1–4 and a redshift for trees 5–8. Comparing the spectra at opposite bias polarities shows that the highest-lying states are probed with negative bias and the lowest-lying ones with positive bias. For positive bias, spin torque drives the electron spin toward the ground state (*m*_*J*_ = −4), making tree 1 the most prominent in the spectra.^38^ In marked contrast, at negative bias, the highest lying states (up to *m*_*J*_ = +3 and +4) are populated, giving tree 8 the largest intensity. The spin pumping is readily visible also in the tunneling spectra (Figure S5), where the saturation at ∼20 pA corresponds to one tunneling electron per ∼8 ns. The IETS step height (Figure 1f) shows that ∼15% of the tunneling electrons relax the atomic spin, which yields an estimate of the intrinsic spin relaxation time *T*_1_ ≈ 50 ns. This relaxation time is comparable to that of other open-valence-shell adatoms on the MgO film^18^ but much shorter than closed-valence-shell Er atoms on the same film.^24^

The hyperfine coupling constant *A* is given approximately by the hyperfine peak spacing seen in the spectra, which is ∼300 MHz for ^153^Eu-b and ∼600 MHz for^151^Eu-b (Figure 2). Fitting the Hamiltonian (eq 1) to the spectra yields hyperfine couplings *A* = 291 ± 13 MHz for ^153^Eu-b, and *A* = 670 ± 13 MHz for ^151^Eu-b (Figure 3b). These hyperfine interactions are smaller by a factor of about two than those of the ions in the gas phase,^39^ likely because the *s*-shell spin density is reduced by delocalizing over neighboring surface atoms or by hybridization with the Eu 5*d* orbitals. Despite this reduced hyperfine magnitude, the measured ratio of *A* for the isotopes on MgO (*A*_151_/*A*_153_ = 2.30) agrees well with the ratio of 2.25 reported for gas-phase Eu^+^ ions^39^ and with the ratio of 2.27 for their nuclear magnetic moments.^39^ We assigned the isotope of each atom based on its relative hyperfine coupling.

We determined the *g-*factor by taking the mean of two symmetrically positioned hyperfine peaks, which approximately eliminates the effects of the hyperfine and CF interactions. Extrapolation of this mean to zero current to eliminate most of the effect of tip fields (Figure S7) yields a *g*-factor of ∼1.97 for both isotopes, in good agreement with IETS measurements (Figure S2).

To understand the magnetic anisotropy, we modeled the effect of orienting the CF axes corresponding to *D* and *E* (eq 2) along different symmetry axes of the bridge adsorption environment (C_2v_ symmetry). Given that our applied magnetic field **B** is oriented nearly in the plane of the surface (Figure 1d), we find that the measured spectra are consistent only with the anisotropy axis oriented along the surface normal, with *D* = +1.52 ± 0.15 GHz, where the sign of *D* indicates easy-plane (hard-axis) anisotropy (Figure S8). A transverse term *E* = +0.36 ± 0.10 GHz oriented along the Mg–Mg bridge direction significantly improves the fit to the data (Figures 3b and S9, Supporting Information Section 3). We note that similar CF parameters are typically found for Gd(III) ions in molecular spin labels, where *D* ≈ 0.5–2 GHz and *E* ≈ 0.1–0.4 GHz.^40^ This similarity is reasonable in light of the 4*f*^7^ electron configuration of Gd(III), whose *f* shell is isoelectronic with Eu^+^. The vanishing orbital angular momentum in 4*f*^7^ ions has been demonstrated to suppress decoherence from spin–lattice relaxation.^41^ Specially designed Gd(III) compounds show promise as candidate spin qubits^42,43^ and spin *qudits*,^36,44,45^ which are *d*-dimensional (*d* > 2) quantum systems being explored as quantum computing platforms.^46,47^ A crucial advantage of 4*f*^7^-based spin qudits is that the entire electron spin manifold can be accessed in typical ESR frequency ranges.^45^ The Eu^+^ studied here can be viewed as a *S* = 4 non-Kramers analogue to the Gd(III) *S* = 7/2 Kramers ion. The additional degrees of freedom due to the *I* = 5/2 nuclear spin of Eu result in a *d* = 54 combined electron–nucleus qudit manifold that may serve, like Gd(III), as a model high-dimensional single-ion qudit.

### Samarium Spin Excitations

**Figure 4:**
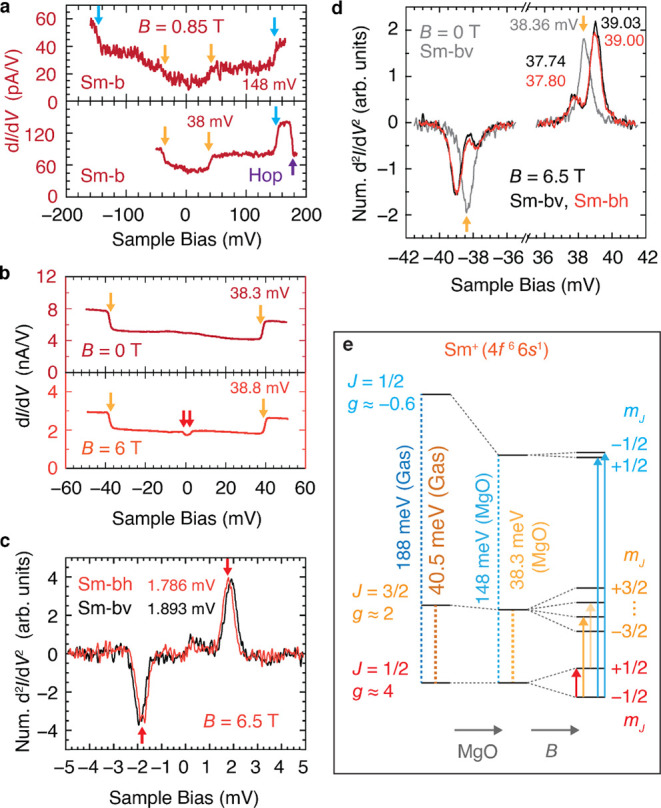
Excitations of samarium. (a) Tunneling spectra (d*I/*d*V*) of bridge-site Sm on MgO (Sm-b). Conductance steps show excitations at ∼148 and at ∼38 mV. Top: low conductance (tip-height set point *I*_set_ = 2.5 pA at *V*_set_ = 80 mV); bottom: higher conductance (5 pA at 80 mV), where the Sm atom hops as indicated due to larger voltage and current, limiting the accessible voltage range. (b) Spectra of Sm-b showing ∼38 mV step at 0 T and at 6 T. The excitation shifts with magnetic field, and a low-energy excitation is visible at 6 T. (c) Low-energy tunneling spectra of bridge-site Sm showing Zeeman splitting of the ground state doublet. Energies as labeled (uncertainty 0.004 mV) for Sm-bh (red) and Sm-bv (black). Tip-height set point 200 pA at 10 mV, *V*_mod_ = 0.2 mV rms. (d) Tunneling spectra near the ∼38 mV excitation of Sm-b with energies in mV as labeled. Spectra are at *B* = 0 (gray) and at *B* = 6.5 T, black for Sm-bv and red for Sm-bh. At 6.5 T the excitation splits into peaks of different amplitudes. The lower energy (and lower amplitude) peak is an excitation beginning from *m*_*J*_ = +1/2, which is an excited state. Set point 50 pA at 10 mV, *V*_mod_ = 0.2 mV rms. Effect of the bridge orientation (Sm-bv versus Sm-bh) is negligible. Spectra in (c) and (d) are d^2^*I*/d*V*^2^ obtained numerically from measured d*I/*d*V* (Figure S11). (e) Schematic energy level diagram of gas-phase and MgO-adsorbed Sm (Sm-b). Application of a magnetic field *B* (right) splits the states. Vertical arrows show transitions assigned in the measured spectra of Sm-b: *m*_*J*_-changing (red), *L*-*S*-tilt from the ground state (orange) and from an excited state (yellow), and 6*s*-flip (blue).

For bridge-site samarium (Sm-b), we observe two excitations in the tunneling spectra, at ∼38 mV and ∼148 mV (Figure 4a,b). The magnetic field splitting of the 38 mV excitation indicates its magnetic origin (Figures 4d and S11). Comparison to gas-phase spectra of Sm ions^35^ yields an assignment of Sm-b to the singly charged cation Sm^+^ with ground-state configuration of 4*f*^6^5*d*^0^6*s*^1^ and electronic level ^8^F_1*/*2_ (*S*_4*f*_ = 3, *L*_4*f*_ = 3, *J*_4*f*_ = 0, *S*_val_ = 1*/*2, *S*_tot_ = 7*/*2 and *J*_tot_ = 1*/*2). It differs from Eu^+^ in having one fewer *f* electron, which endows it with a large orbital angular momentum *L* = 3. The first excitation of the free ion is at 40.5 mV, a transition to ^8^F_3*/*2_ that excites the *f*-shell from *J*_4*f*_ = 0 to 1 by “tilting” *L* with respect to *S*, to which we assign the observed 38 mV transition (Figure 4e). The next higher excitation of the free ion that is compatible with inelastic tunneling selection rules (Δ*m*_*J*_ = ±1 or 0) occurs at 188 mV and excites from the ground state to the ^6^*F*_1/2_ multiplet, where *S*_4*f*_ and *S*_val_ become antiparallel in a 6*s*-flip, similar to Eu^+^ (Figure 4e). For the surface adsorbed species Sm-b we detect this transition at ∼148 mV, which is similar to the gas-phase value, but smaller for the reasons discussed for Eu^+^. For oxygen-site Sm (Sm–O), the “*L-S-*tilt” excitation splits into two *J* = 3/2 doublets, at 26.2 and 88.8 mV, because of strong magneto-crystalline anisotropy (Figure S12). In a magnetic field, Sm-b exhibits a low-energy spin-flip excitation, at ∼1.8 mV for *B* = 6.5 T (Figure 4c). This excitation occurs at energies that correspond to *g* = 4.75 ± 0.03 for the horizontally oriented bridge site (Sm-bh) and *g* = 5.03 ± 0.04 for the vertically oriented bridge site (Sm-bv). For Sm–O, it occurs at 1.55 mV at 6 T, giving *g* = 4.46 ± 0.03 (Figure S12). These *g*-factors are notably higher than both the Sm^+^ gas-phase ion, for which *g* = 3.950,^35^ and the calculated Landé *g*-factor of 4 (given *L* = 3 and *S* = 7/2). These *g*-factors are extraordinarily high for atomic spins, and they arise from the large but nearly canceling orbital and spin angular momenta within the atom (Figures S13a, S14).

Sm and Eu are the first lanthanide atoms known to exhibit inelastic tunneling excitations when adsorbed on MgO/Ag(001), in contrast to the featureless spectra seen previously for Dy, Ho, and Er on this surface.^3,24,32^ We note that these tunneling spectra closely resemble the spectra reported for Sm and Eu on graphene on Ir(111).^28^ When adsorbed on the Mg sites of MgO, in contrast, both Sm and Eu exhibit featureless d*I/*d*V* spectra (Figures S2f and S12), which we propose is the result of a change in charge state compared to the bridge and O sites, and consequent pairing of the valence-shell electrons.

**Figure 5:**
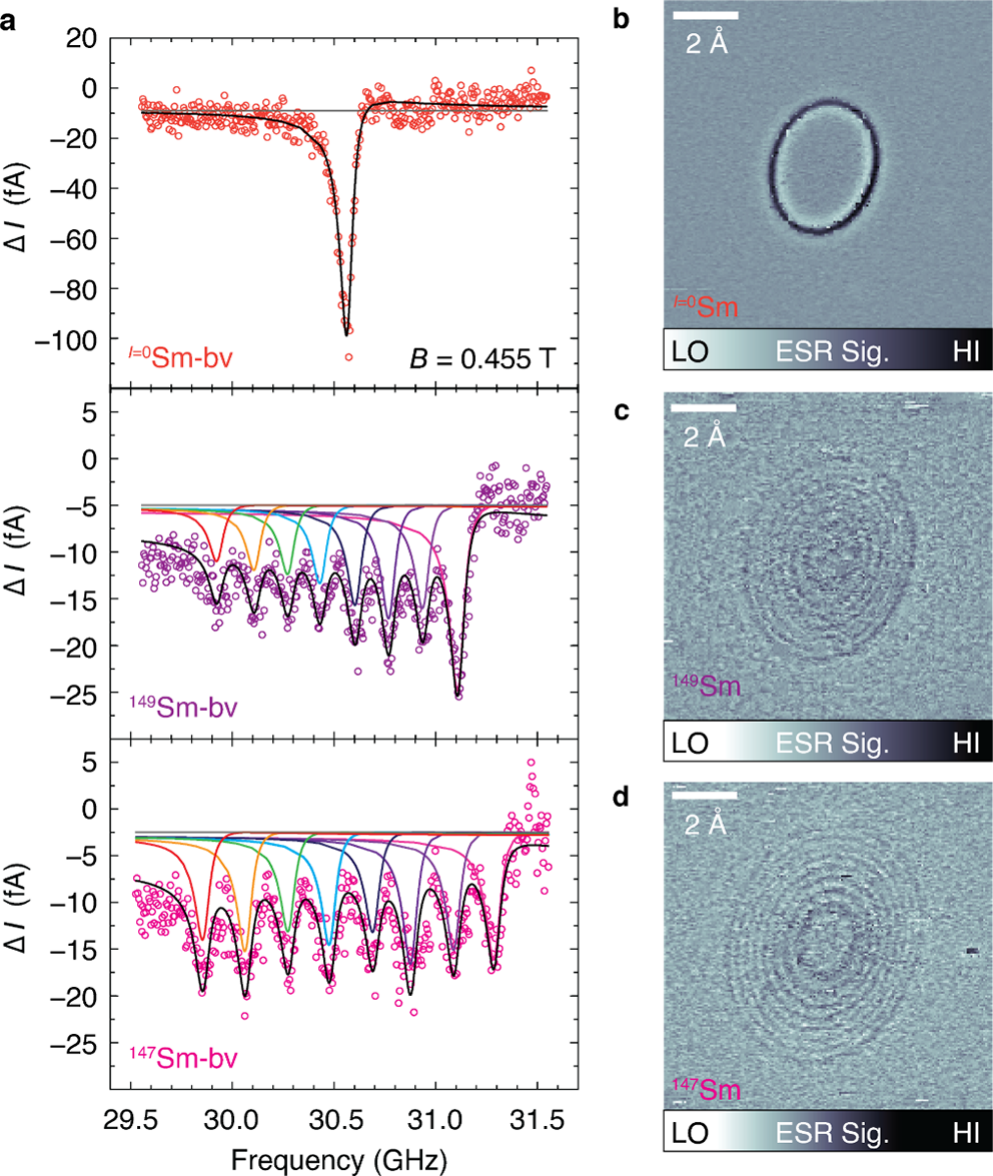
ESR spectra and magnetic resonance images of Sm isotopes. (a) ESR spectra of different Sm isotopes: Zero-nuclear-spin isotope of Sm-b (*I* = 0, 71.19% total for ^144,148,150,152,154^Sm), ^149^Sm-b (*I* = 7*/*2, 13.82%), and ^147^Sm-b (*I* = 7*/*2, 14.99%). All spectra: tip-height set-point 20 pA at 25 mV, *V*_RF_ = 5 mV, *T* = 0.6 K, *B* = 0.455 T, Sm at a vertical bridge site of MgO (Sm-bv). (b–d) Magnetic resonance images show concentric rings where the tip field *B*_tip_ shifts the atom into resonance at *f*_RF_ = 29.0 GHz. The *I* = 0 and *I* = 7/2 isotopes show one and eight rings, respectively. Set point 80 pA at 25 mV, *V*_DC_ = −15 mV, *V*_RF_ = 20 mV.

The most abundant Sm nuclear spin is *I* = 0 for five isotopes, which show a single ESR peak (Figure 5a, top). The two remaining isotopes have *I* = 7*/*2 so they display eight hyperfine peaks (Figure 5a, bottom). From fitting these spectra, we derive *A* = 170 ± 6 MHz for ^149^Sm-b and *A* = 205 ± 8 MHz for ^147^Sm-b. The ratio of these two hyperfine couplings (*A*_147_/*A*_149_ = 1.21) matches the ratio of their nuclear magnetic moments (1.21),^48^ suggesting that the different nuclear moments are the origin of the different hyperfine parameters. Magnetic resonance images of individual Sm atoms each show concentric resonant slice rings, one for each nuclear spin state (Figures 5b–d and S15), which provides visualization of the nuclear spin states and the tip-adatom magnetic interaction.^49^

We expect the magneto-crystalline anisotropy for adsorbed Sm^+^ to be large because of its large orbital angular momentum *L* = 3, as exhibited by the large splitting of the *L-S*-tilt excitation in Sm–O (Figure S12). The ground-state multiplet of Sm is a Kramers doublet, having *m*_*J*_ = ±1*/*2, so it cannot exhibit zero-field splitting. It can, however, exhibit anisotropy in the *g*-factor. Comparing the two Sm bridge orientations, ESR spectra yield effective *g*-factors of 4.71 ± 0.05 and 5.04 ± 0.08 for Sm-bh and Sm-bv, respectively (Figure S14), in agreement with the values obtained above from high-field tunneling spectra. This pronounced *g*-factor difference between sites implies a large in-plane anisotropy of the *g*-tensor, considering that our magnetic field is applied ∼42° away from the O–O axis of Sm-bh, which differs only slightly from the ∼48° angle to the Sm-bv axis (Figure 1d). This large in-plane *g*-tensor anisotropy may be useful for direction-dependent magnetic field sensing using Sm-b as a single-atom magnetometer.

## Conclusions

The Eu^+^ and Sm^+^ studied here constitute an unusual case of stable, open-shell lanthanide cations in a solid-state system. The key ingredients for realizing the open-shell state in this work include the greater propensity for Eu and Sm to be divalent in compounds, compared to other lanthanides,^30^ and the electron transfer from each atom to the metal substrate through the thin insulating film. This electron transfer is well-known for adsorbates on metal–supported MgO thin films^21,22,50,51^ and a variety of other insulating films on metal substrates.^52,53^ We therefore propose such adsorption and charging as a general strategy for preparing uncommon monovalent lanthanide species for use in atomic-scale devices (see Supporting Information Section 8).

Our demonstration of ESR-STM on monovalent lanthanides opens electronic access to these versatile elements one atom at a time and allows the use of their magnetic quantum properties, which are tailored by choosing their local atomic environment. With a single ESR spectrum, we determine the isotope, hyperfine interactions, and magneto-crystalline anisotropy. We anticipate that the relatively large hyperfine coupling in these atoms will enable direct control of their nuclear spins^19^ with potentially much longer lifetimes than for electron spins.^4^ The unpaired valence electrons are expected to facilitate stronger magnetic coupling between the atoms, enabling their use in engineered atomic-scale magnetic nanostructures.^54^ Recently demonstrated remote driving methods^55^ will additionally lengthen relaxation times, allowing quantum gate operations on precisely positioned coupled lanthanide spins.

## Methods

### Microscope and Sample

Measurements were performed in a home-built ultrahigh-vacuum STM operating at 0.6–1.2 K as noted. An MgO thin film was grown on an atomically clean Ag(001) single crystal held at ∼340 °C, by thermally evaporating Mg from a Knudsen cell in a 1 × 10^–6^ Torr O_2_ environment at ∼0.5 monolayer (ML) per minute for an average coverage of ∼1.6 ML MgO. This produced two-ML MgO terraces interspersed with clean Ag(001) regions used for tip preparation. Metal atoms of Fe, Ti, Eu, and Sm were deposited one element at a time by e-beam evaporation from pure metal pieces onto the sample held at ∼10 K. The lanthanide evaporators were prepared in a nitrogen glovebox to avoid corrosion. Previous studies indicated that the Ti atoms may be hydrogenated (TiH),^14−18,20^ and we describe them here as Ti for simplicity. An external magnetic field was applied at an angle ∼9° out of the surface plane with the in-plane component aligned nearly along the [100] direction of the MgO lattice, as described in the text. STM images were acquired in constant-current mode and all voltages refer to the sample voltage with respect to the tip. Voltages and energies are treated as interchangeable, where the factor of the elementary charge e is implied when giving energies in units of mV.

### ESR Apparatus

The ESR spectra were acquired by sweeping the frequency of an RF voltage generated by an RF generator (Agilent E8257D) across the tunneling junction and monitoring changes in the tunneling current. The signal was modulated at 337.11 Hz by chopping *V*_RF_, which allowed readout of the spectrum from the measured tunnel current (room-temperature electrometer model FEMTO DLPCA-200) applied to a lock-in amplifier (Stanford Research Systems SR830). The RF and DC bias voltages were combined at room temperature using an RF diplexer and carried to the STM tip through semirigid coaxial cables with a loss of ∼30 dB at 20 GHz.

### Applied Magnetic Field

The field was applied nearly in-plane as shown in Figure 1d, a direction chosen to allow ESR of both Fe and of spin-1/2 species in previous experiments. The field was oriented at nearly equal angles to the bridge-site orientations, but the slight (∼3°) deviation from the high-symmetry angle allowed us to distinguish the anisotropy parameters for each orientation. The uncertainties in our given *g*-factors are the consequence of spectral peak fitting uncertainties, added to a 0.5% uncertainty in the applied magnetic field.

### STM Tips

The STM tip was a mechanically sharpened bulk iridium wire that was presumably coated with silver by indentation into the sample. It was prepared in vacuum by field emission and by indentations into the Ag sample until the tip gave a good lateral resolution in STM images. All tips used the same bulk Ir wire but differed in the elemental identity and precise arrangement of atoms near the apex. To prepare a spin-polarized tip, about 2–8 Fe atoms were each transferred from the MgO onto the tip by applying a bias voltage (∼0.55 V) while withdrawing the tip from near point contact with the Fe atom. The ESR spectra shown in the main text were recorded with “Tip 1”, an STM tip apex that yielded unusually high ESR contrast on ^48^Ti-b atoms, where Δ*I/I* was about 9% as shown in Figure S5, compared to ∼2–4% observed for typical spin-polarized tips used for ESR. This tip produced spin-torque in the Eu-b electron spin, and showed strong bias-voltage dependence for tunneling spectra of Ti–O, which indicates spin polarization (Figure S5). It was obtained as for other ESR tips by picking up several Fe atoms onto a presumably Ag-coated tip. To illustrate the high signal-to-noise ratio obtained with this particular tip, it was used to acquire ESR spectra of bridge-site Ti on this surface (Figure S4). All ESR data were acquired using Tip 1 except where noted. It was used to acquire all ESR data shown in the main text Figures 2, 3 and 5 and S3, S4, S6, S7, S14d–g and S15.

### Atom Manipulation

Eu and Sm atoms were repositioned on the surface by pulling them with the STM tip while close to point-contact, by using bias *V*_DC_ = 10 mV and tunnel current *I* = 2–6 nA. Most tips tended to move the atoms to either O or bridge sites preferentially, with bridge-favoring tips being much more frequent. In contrast it was always possible to position the atoms at the Mg sites by briefly applying |*V* | > 200 mV at ∼20 pA. We speculate that the different binding site preferences for manipulation using different tips may arise from different local work functions (tunneling barrier heights) of each tip apex material and atomic arrangement, and consequently of different electric fields present in the junction, which can readily dominate the bias-voltage dependence of the electric field. Manipulation and binding-site examples are shown in Figure S1.

## Supporting Information Available

Discussion of atom manipulation methods and adsorption sites; data analysis and state assignments; determination of *g*-factors and origin of large *g*-factors; properties of monovalent lanthanides; figures showing raw data and additional data using different STM tips, tunneling conditions, and surface binding sites (PDF)
