# Supplementary material for: Direct Electrical Access to the Spin Manifolds of Individual Lanthanide Atoms
Source: ACS Nano. 2025 Jan 14;19(3):3705–13. doi: 10.1021/acsnano.4c14327 (PMC11781023; doi:10.1021/acsnano.4c14327)
Supplement: Supplementary file 1 — nn4c14327_si_001.pdf [file nn4c14327_si_001.pdf]

Supporting Information for

Direct Electrical Access to the Spin Manifolds of Individual Lanthanide Atoms

Gregory Czap[1], Kyungju Noh[1,2,3], Jairo Velasco Jr.[1,4], Roger M. Macfarlane[1], Harald Brune[1,5*] and Christopher P. Lutz[1*]

[1]IBM Almaden Research Center, 650 Harry Road, San Jose, CA 95120, USA

[2]Center for Quantum Nanoscience (QNS), Institute of Basic Science (IBS), 03760 Seoul, Republic of Korea

[3]Department of Physics, Ewha Womans University, 03760 Seoul, Republic of Korea

[4]Department of Physics, University of California, Santa Cruz CA 95064, USA

[5]Institute of Physics, École Polytechnique Fédérale de Lausanne (EPFL), CH-1015 Lausanne, Switzerland

[*]Corresponding authors. Email: cplutz@us.ibm.com, harald.brune@epfl.ch

Contents

Supplementary Notes 3
1. Eu and Sm adsorption site stability 3
2. Determination of ground state g-factors 3
3. Modeling Eu magnetic anisotropy 6
4. Nature of the splitting of the 38 mV excitation of Sm-b 8
5. State assignments for the 38 mV excitation of Sm-b 9
6. State assignments for the Sm-O IETS transitions 14
7. Origin of large Landé *g*-factors for Sm 15
8. Stabilizing Ln(I) on surfaces 17
Figure S1. STM images and binding sites for Eu and Sm on MgO film. 21
Figure S2. Additional tunneling spectra of bridge-site Eu (Eu-b). 22
Figure S3. Raw data for ESR spectra of Eu-b shown in main text Figure 2. 25
Figure S4. ESR spectra of Ti isotopes ^{48}Ti-b and ^{49}Ti-b. 26
Figure S5. Spin torque of Eu-b and detection of tip spin polarization using Ti-O. 27
Figure S6. Processing ESR spectra for ^{153}Eu-b shown in main text Figure 3. 29
Figure S7. Determining the *g*-factor of Eu-b from ESR spectra. 30
Figure S8. Calculated states and transitions from model Hamiltonian of Eu-b. 32
Figure S9. Effect of transverse crystal field *E* on model Hamiltonian for Eu-b. 33
Figure S10. Tunneling spectra of Eu at oxygen and bridge sites. 35
Figure S11. Tunneling spectra for Sm at bridge sites. 37
Figure S12. Tunneling spectra of Sm at Mg and oxygen sites. 38
Figure S13. Proposed excitation schemes and calculated Landé *g*-factors for Sm^+ 40
Figure S14. Anisotropic *g*-factor determination from ESR spectra of Sm-b. 41
Figure S15. Post-processing of Sm magnetic resonance image (MRI) data. 43

Supplementary Notes

1. Eu and Sm adsorption site stability

After deposition onto the cold MgO surface, both Eu and Sm could be found on either the Mg-site or bridge site (b-site). However, it was much more common to find Eu on the b-site and Sm on the Mg-site. In addition, it was found to be significantly easier to cause the Sm atoms to hop from a b-site to an Mg-site compared to Eu atoms. Either close tip proximity or elevated bias (or both) caused the atoms to hop to the Mg-site, and the voltage and proximity thresholds for this hopping were consistently lower for Sm-b than for Eu-b. This hopping threshold additionally showed significant variation among tip apexes. It was much more difficult to re-position Sm or Eu to the O-site than to the b-site. The ability to position Sm or Eu on the O-site depended strongly on the particular tip apex used, with certain rare tip apexes capable of repositioning Sm to the O-site reproducibly. We encountered only one tip apex that was consistently able to reposition Eu to the O-site. This may imply that both atoms have a shallow barrier to diffusion from the O-site.

2. Determination of ground state g-factors

The *g*-factors were determined for Eu-b and for Sm-b by two methods: by using IETS with a non-spin polarized tip, and by ESR spectra using a magnetic tip. In-plane *g*-factor anisotropy is measured by comparing the two bridge-site orientations. Our external field is applied ~42° with respect to the h-bridge direction (~48° to the v-bridge direction). This alignment is a holdover from previous experiments in which we nominally aligned the field to 45°, which would make the two bridge-site orientations indistinguishable. An ~3° sample miscut and mounting imperfection away from 45° allows us to distinguish the *g*-factors for different bridge site orientations. Future measurements using a rotatable magnetic field or different sample orientation should allow more precise determination of in-plan anisotropy parameters.

Although the magnetic anisotropy for Eu-b is expected to be very small because of its lack of orbital angular momentum, to explore the possibility of *g*-factor anisotropy we acquired IETS

spectra for both Eu-bh and Eu-bv (Figure S2a,b). The measured Zeeman energies at $B = 6.5$ T for the two species are identical within the uncertainty, such that we resolve no *g*-factor anisotropy. The Zeeman energy of both Eu-b orientations implies a *g*-factor 1.970 (mean of 1.977 for Eu-bh and 1.964 for Eu-bv), close to the Eu^+ gas phase value 1.984.

For Sm-b, a similar comparison of the 6.5 T tunneling spectra between the two orientations, Sm-bh and Sm-bv, using a non-spin polarized tip is shown in main text Figure 4c and Figure S11a. In contrast to Eu-b, Sm-b shows significant *g*-factor anisotropy as evidenced by the > 0.1 mV difference in the Zeeman splitting between the two orientations. These spectra give apparent *g*-factors of 4.747 for Sm-bh and 5.031 for Sm-bv for this applied direction for *B*. Uncertainties are in these *g*-factors are 0.7%, which includes 0.2% peak-fit uncertainty and 0.5% uncertainty in the applied magnetic field.

To obtain reliable *g*-factors from ESR, we must remove the effect of the tip magnetic field to the extent possible on the measurement since this magnetic field is unknown and uncharacterized for each tip apex. We compensate for the effect of the exchange interaction with the tip by extrapolating tip-height-dependent peak energies to zero tunnel current (Figures S7 and S14 for Eu-b and Sm-b, respectively) such that only the externally applied magnetic field *B* is expected to act on the atom, and the effect of B_{tip} is expected to be largely eliminated or reduced to a long-range magnetic dipole component. For Eu-b in particular, in order to remove in addition the effect of the CF we take the average energies of ESR peaks that have opposite-sign CF shift and opposite-sign hyperfine shifts. These are found by identifying the symmetrically opposite hyperfine peaks in trees 1 and 8, which are found at opposite bias (Figure S7c,f). Since the bias voltages are chosen symmetrically around zero bias, this may also remove small but systematically observed Stark shifts caused by the bias voltage. For ^{151}Eu-bh, this yields $E_{Zee} = 23.52 \pm 0.05$ GHz at 0.85 T, or $g = 1.977 \pm 0.004$. For ^{153}Eu-bv we find $E_{Zee} = 23.36 \pm 0.05$ GHz at 0.85 T, which yields a nearly identical value of $g = 1.964 \pm 0.004$. (These uncertainties do not include the 0.5% uncertainty in the applied magnetic field, which should result in the same systematic error for both orientations.) Both values are close to that obtained from IETS spectra (1.966) and to the gas phase value for Eu^+ (1.984). A similar comparison, for Sm-bh and Sm-bv, made with the same tip apex is shown in Figure S14d–g.

For a given tip, the *g*-factors of both elements measured by ESR were found to be systematically either higher or lower than the *g*-factors derived from tunneling spectra (conductance steps measured using non-spin-polarized tips). The ESR measurements require a spin-polarized tip, making exchange coupling to the tip and dipole magnetic fields from the tip unavoidable. The distance-dependent spectra show that the dominant tip-sample interaction is linear with tunnel current, and therefore of exchange character. Extrapolating to zero tunnel current removes the effect of exchange interaction, but the small dipolar magnetic field of the tip is not removed by extrapolating to zero current because it decays too slowly as the current is reduced. The tip magnetic dipole moments are expected to vary significantly among different tip apexes owing to the random, uncontrolled nature of each Fe tip preparation, which may explain the significant tip-dependent variations in Zeeman energy found for Sm-bh and Sm-bv species. This stray tip magnetic field is additive with the external magnetic field. The effects of this uncompensated dipole field are illustrated by the variation among Sm-bh and Sm-bv *g*-factors derived from ESR using different tips as shown in Figure S14. The lateral tip position can also influence the tip field sensed by the atom, despite extrapolating to the limit of zero tunneling current, as shown in Figure S14a. Nevertheless, Sm-bv was always found to have a higher resonant frequency than Sm-bh, as for Ti-bv compared to Ti-bh. Overall, despite variations in tips, the apparent *g*-factors for Sm-bh and Sm-bv obtained from ESR measurements (Figure S14) differed by no more than 2% from the values obtained from IETS (Figure 4c and Figure S11a).

Interestingly, we also observed strong ESR signal intensity variation between Sm-bh and Sm-bv, which depended on the tip apex. "Tip 1" and "Tip 3" data showed stronger signal intensity for Sm-bv compared to Sm-bh (for "Tip 3" the difference was a factor of 5 in amplitude), while "Tip 2" and "Tip 4" instead showed stronger signal intensity for Sm-bh. Signal intensity differences between Ti-bh and Ti-bv could also be observed, but the relative change in intensity between the two species of Ti was much less compared to the difference for Sm. We note that in the data shown here, the intensity variations between Ti and Sm qualitatively follow each other (*i.e.* when Sm-bh has a more intense signal than Sm-bv, Ti-bh similarly has a more intense signal than Ti-bv, and vice-versa). We speculate that this asymmetry may be related to the in-plane components of the

tip dipole field, which may provide greater (or lesser) driving strength for one bridge orientation or the other due to the in-plane g-factor anisotropy of Sm-b and Ti-b.

We observed that with most tips, the ESR signal for Sm-b appeared as a "dip" (decrease in conductance) at positive bias, similar to Eu-b, while ESR signals for Ti-b and Ti-O appeared as "peaks" (increase in conductance) under similar conditions when the same tip apex was used. This consistent observation suggests opposite-sign tunnel magnetoresistance for the lanthanides compared to Ti.

To determine the g-factor of the Eu-b multiplet at ~163 mV (the 6s-flip excitation) we analyze the measured shift in the excitation as the field is changed from 0 to 6.5 T, which is 0.7 ± 0.1 mV (Figure 1e). We model the shift as the difference in initial and final Zeeman energies $g_f \mu_B B m_f - g_i \mu_B B m_i$, where $m_i = -4$, $m_f = -3$ (the only transition from the ground state allowed by $\Delta m_J = \pm 1$ or 0), $g_i = 1.97 \pm 0.01$ is the ground multiplet g-factor, and $\mu_B B = 0.3762$ at 6.5 T. This yields an excited state g-factor $g_f = 2.0 \pm 0.1$, which is consistent with the gas-phase Eu^+ ion's value of 1.981 for this excited state.

3. Modeling Eu magnetic anisotropy

To understand the effect of the magnetic anisotropy on Eu-b expressed by the crystal field terms in the Hamiltonian (main text Eqs. 1 and 2, illustrated in Figs. S8 and S9), we first consider the effect of a hypothetical purely axial crystal field DS_z^2 aligned to the magnetic field. This anisotropy would result in a quadratic dependence of the energy on S_z, and consequently a linear dependence of the ESR frequency on the initial-state quantum number m_J, as observed to good approximation in the spectra and depicted in Figure S8b. However, the field direction is not aligned to any of the symmetry axes of the MgO bridge site in our experiment (main text Figure 1d). Assigning the Hamiltonian principal anisotropy axis to an in-plane symmetry direction X or Y yields a very poor fit to the spectra (Figure S8d), ruling out this possibility. In contrast, an out-of-plane hard-axis anisotropy yields the nearly linear spacing observed (Figure S8c). Assignment of hard-axis anisotropy in the out-of-plane direction is the only assignment that satisfies the known geometric

symmetry and peak-height ordering that we observe. A qualitative fit yields an out-of-plane crystal field parameter of $D \approx 1.5$ GHz ≈ 6.2 μeV. This crystal field parameter is very small compared to other surface-adsorbed lanthanides[32], a consequence of Eu having zero orbital angular momentum. Simulated spectra shown in Figure S8b–d neglect the hyperfine coupling for simplicity, while the spectra shown in Figure S8e uses hyperfine coupling $A = 700$ MHz, which is qualitatively consistent with ^{151}Eu. One can then compare Figure S8e with the ^{151}Eu spectra shown in main text Figure 2c,d. The corresponding energy eigenvalues are shown in Figure S8f while excitation energies are shown in Figure S8g, both as a function of B. At low B, the magnetic anisotropy D, Zeeman energy and hyperfine coupling A all become comparable in energy scale, resulting in complex changes to the excitation spectrum.

Since Eu-b binds to the bridge site with C_{2v} symmetry, transverse magnetic anisotropy is also expected. This situation is depicted schematically in Figure S9a. The inclusion of the transverse anisotropy parameter E changes the apparent separation in excitation energies between trees (different electron spin excitations). Simulated spectra for a Hamiltonian assuming transverse anisotropy $E = 0$ are shown in Figure S9b, and the inclusion of finite E approaching the upper allowed value $E = \frac{1}{3}D$ is shown in Figure S9c,d for two different orientations of external magnetic field relative to the transverse anisotropy axis E. A key indicator of the presence of transverse anisotropy is its effect on the apparent crystal field splitting (spacing between trees) for Eu-bh compared to Eu-bv. Depending on whether E is oriented along the O-O or Mg-Mg direction, following Figure S9c,d, numerical modeling indicates that one orientation of Eu-b has an overall larger apparent spacing between trees and the other Eu-b orientation has overall smaller spacing. This transverse anisotropy also has the effect of expanding the excitation energy spacing between trees 1 and 2 relative to trees 7 and 8, as compared to the case where no transverse anisotropy is present. ESR measurements confirm these adsorption-site dependencies on the tree spacing as shown in Figure S9e–i. We find the transverse parameter $E \approx 350$ MHz = 1.45 μeV.

We emphasize that our experimental uncertainty in the total magnetic field direction, which arises from tip apex-dependent variations in the dipole field contribution from the tip, prevents a quantitative fit of D and E. In addition, since a proper fit of these parameters necessarily requires data acquired at both positive and negative bias (to fit especially Trees 1, 2, 7 and 8 to the model

Hamiltonian excitation energies), there remains the possibility of Stark shifts which may reposition Trees 1 and 2 relative to 7 and 8 which are not accounted for in the model Hamiltonian. These Stark shifts have been reported recently to introduce significant changes to the tip-adsorbate exchange field[20] and are hinted at in data acquired for Sm shown in Fig. S14e. Accordingly, the experimental uncertainties provided in the main text for D and E are only a rough guide which reflects the typical range of values observed upon perturbing the direction of the magnetic field in the model by a few degrees in each direction. A more quantitative characterization of the Eu crystal field parameters would require a multi-axis magnetic field.

4. Nature of the splitting of the 38 mV excitation of Sm-b

This section provides evidence that the splitting of the ~38 mV IETS peak seen for Sm-b at high magnetic field B arises from heating of the field-split ground-state multiplet, not from transitions from the ground state to multiple final states. Consequently, only a single transition is observed when starting from a given state ($m_J = \pm\frac{1}{2}$) in the ground multiplet ($J = \frac{1}{2}$).

As shown in main text Figure 4d and in Figure S11b,f,h, the 38.4 mV excitation splits at high magnetic field $B = 6.5$ T into a strong 39.0 mV peak and a weaker 37.8 mV peak (values are averages from Figure 4d). These high-field peaks are spaced symmetrically about the 38.4 mV zero-field peak. Based on the similarity of the energy to the 40.5 mV free Sm+ excitation, we assign the peaks to different allowed "*L*-*S*-tilt" excitations, in which $J = \frac{1}{2} \rightarrow J = \frac{3}{2}$. The intensity of the smaller peak varied dramatically as a function of tunnel current setpoint used during data acquisition (Figure S11c). Although it is plausible that IETS selection rules vary depending on the tip proximity to the *f*-shell electrons, we propose that the much more likely explanation is that the tunnel current heats the Sm-b spin from the magnetic ground state to populate both of the $m_J = \pm\frac{1}{2}$ states through random IETS spin-flip events. The Zeeman energy required to flip the Sm-b from the ground state to the first excited state, ($m_J = -\frac{1}{2} \rightarrow +\frac{1}{2}$), is much lower in energy than the 38 mV *L*-*S*-tilt excitation. For that reason, use of bias voltages near 38 mV to observe that transition randomize the Sm-b ground multiplet through spin-flip IETS events to equally populate $m_J = -\frac{1}{2}$

and $m_J = +\frac{1}{2}$, in the limit of large current. The precise ratio depends on the tunnel current, the lifetime of the $m_J = +\frac{1}{2}$ excited state, and the tip spin polarization, which we believe to be near zero. As the time-average population of $m_J = +\frac{1}{2}$ increases, *L*-*S*-tilt excitations increasingly correspond to $\left|\frac{1}{2}, +\frac{1}{2}\right\rangle \rightarrow \left|\frac{3}{2}, +\frac{1}{2}\right\rangle$, and reduce the dominant $\left|\frac{1}{2}, -\frac{1}{2}\right\rangle \rightarrow \left|\frac{3}{2}, -\frac{1}{2}\right\rangle$ transitions from the ground state. Consequently, as the tunneling current increases, the intensity of the 37.8 mV transition increases, so that the ratio of the 39.0 mV IETS signal to the 37.8 mV IETS signal decreases. This ratio is plotted in Figure S11d and shows an exponential dependence on the tunneling current. We cannot at present explain why the ratio appears to converge to ~1.5 rather than 1 as expected for an equally-randomized ground multiplet population expected for a non-spin-polarized tip[38]. (A similar weaker, lower energy peak was observed for the 26.2 mV excitation of Sm-O as seen in Figure S12g–j). From these observations we conclude that the 37.8 mV excitation arises from tunneling electrons heating the ground state. Consequently, there is only one observed *L*-*S*-tilt transition for each initial state ($J = \frac{1}{2}$ to $\frac{3}{2}$), and the two observed peaks arise from occupation of two initial states in the ground multiplet.

5. State assignments for the 38 mV excitation of Sm-b

Here we consider alternative interpretations of the observed excitations seen in tunneling spectra at ~38 mV for Sm-b, which is the excitation energy required to tilt *L* with respect to *S* that we term the "*L*-*S* tilt" excitation. When starting from the $J = 1/2$, $m_J = -1/2$ ground state, one might expect transitions to any of the four states in the $J = 3/2$ excited state multiplet, subject to any selection rules for such transitions. We consider how the measured excitation energy shifts in a magnetic field, and interpret the *g*-factors implied by this shift for each case in order identify the likeliest value of m_J for the final state.

In the following we make use of the measured excitation energies $\Delta E_0 = 38.36$ mV measured by IETS at $B = 0$ T, and a field-shifted energy $\Delta E_{6.5\ \mathrm{T}} = 39.03$ mV measured at $B = 6.5$ T (Figs. 4D,

S11b). This gives a net Zeeman shift $\Delta E_{6.5\,\mathrm{T}} - \Delta E_0$ = 0.67 mV. (We disregard the weaker peak that shifts in a magnetic field by an equal amount downward, to 37.74 mV (Figs. 4D, S11b), by attributing it to the excitation from the $\left|\frac{1}{2}, +\frac{1}{2}\right\rangle$ state. This initial state is occupied only due to heating of the ground-state multiplet, as discussed in section 4.)

Small anisotropy case. We first consider the case where *D* is significantly smaller than the Zeeman energy at large magnetic field, in which case the *J* = 3/2 spin is oriented by the external magnetic field like a free spin. In this situation, the excitation energy for a transition $|J_i, m_i\rangle \rightarrow |J_f, m_f\rangle$ is given by the sum of the zero-field excitation energy ΔE_0 and the change in Zeeman energy:

$$\Delta E = E_f - E_i = \Delta E_0 + g_f \mu_B B m_f - g_i \mu_B B m_i \qquad \text{(Eq. S1)}$$

where g_i and g_f are the *g*-factors for the initial and final states, and μ_B is the Bohr magneton. These *g*-factors are effective *g*-factors under the conditions of our applied field direction, which is not along any of the high-symmetry directions of the binding site, so we expect that they may fall somewhere in the range spanned by the *g*-factors for each axis in the presence of the crystal field. Since we lack measurements in a vector magnetic field, knowledge of the axis-dependent *g*-factors is beyond the scope of this analysis. Solving for the final state *g*-factor gives

$$g_f = \frac{\Delta \mathrm{E} - \Delta \mathrm{E}_0 + g_i \mu_B B m_i}{\mu_B B m_f} \qquad \text{(Eq. S2)}$$

We consider transitions $\left|\frac{1}{2}, -\frac{1}{2}\right\rangle \rightarrow \left|\frac{3}{2}, m_f\right\rangle$ for each possible value of $m_f = -\frac{3}{2}, -\frac{1}{2}, +\frac{1}{2}, +\frac{3}{2}$. Using $\mu_B B = 0.2376$ mV at *B* = 6.5 T, and the measured initial-state *g*-factor $g_i = g_{\frac{3}{2}} = 5.03$ (Figure 4 and Figure S11, choosing values that apply to Sm-bv), Eq. S2 yields respective final-state *g*-factors $g_{\frac{3}{2}} = 0.49, 1.47, -1.47, -0.49$. The negative *g*-factors preclude $m_f = +\frac{1}{2}$ and $+\frac{3}{2}$ as not physically plausible. The *g*-factor of 0.49 is too small to be likely, but the *g*-factor of 1.47

is plausibly consistent with gas phase value 1.975. It corresponds to the transition $\left| \frac{1}{2}, -\frac{1}{2} \right\rangle \rightarrow \left| \frac{3}{2}, -\frac{1}{2} \right\rangle$, and the absence of other visible excitations implies the selection rule $\Delta m_J = 0$. Consequently, under the assumption that magnetic anisotropy energy of Sm-b is much lower than the Zeeman energy at high field, we consider this transition to be the likeliest assignment of the ~38 mV transition of Sm-b. However, we do not have reason to expect that the anisotropy is smaller than the Zeeman energy, so we next consider cases in which the magnetic anisotropy energy is large for this multiplet of Sm-b.

Large anisotropy cases: In the schematic shown in main text Figure 4e (reproduced in Figure S13b), we had for simplicity disregarded the zero-field splitting of the J = 3/2 excited state that arises from the substrate crystal field. However, this multiplet has non-zero orbital angular momentum (L = 3) so significant magneto-crystalline anisotropy is possible. This anisotropy is expected to split the J = 3/2 multiplet into an $m_J = \pm\frac{1}{2}$ doublet and a $m_J = \pm\frac{3}{2}$ doublet, as depicted in Figure S13c,d. If we include large magnetic anisotropy with a leading term describing axial anisotropy DJ_z^2, we can no longer use the above simplified expressions (Eqs. S1 and S2) to obtain the $g_{\frac{3}{2}}$ factor. Instead, we consider the Hamiltonian for the Sm electronic spin system:

$$H = g\mu_B \mathbf{J}\cdot\mathbf{B} + DJ_z^2 + \lambda\mathbf{L}\cdot\mathbf{S}_{4f} + J_{IA}\mathbf{S}_{6s}\cdot\mathbf{S}_{4f} \quad \text{(Eq. S3)}$$

where $g\mu_B\mathbf{J}\cdot\mathbf{B}$ is the Zeeman energy of the total moment in the external magnetic field B, DJ_z^2 is the leading crystal field interaction which gives rise to axial magnetic anisotropy, $\lambda\mathbf{L}\cdot\mathbf{S}_{4f}$ is the spin-orbit coupling between the orbital moment L and S_{4f} that gives rise to the L-S-tilt excitation energy, and $J_{IA}\mathbf{S}_{6s}\cdot\mathbf{S}_{4f}$ is the intra-atomic exchange interaction between the 6*s* and 4*f* electrons that gives rise to the "6*s*-flip" excitation. The excitation energy is given by the difference $\Delta E = E_{J=3/2} - E_{J=1/2}$, and its shift with magnetic field is $\Delta = \Delta E_{6.5\ \mathrm{T}} - \Delta E_0$, which must match our measured shift. The measure a shift is Δ = +0.67 eV, obtained from ΔE_0 = 38.36 mV at B = 0 and $\Delta E_{6.5\ \mathrm{T}}$ = 39.03 mV at B = 6.5 T (Figure 4d).

We make the simplifying assumptions that the spin-orbit term $\lambda\mathbf{L}\cdot\mathbf{S}_{4f}$ and the intra-atomic exchange term $J_{IA}\mathbf{S}_{6s}\cdot\mathbf{S}_{4f}$ are essentially field-independent; in other words, they give the same offsets to ΔE_0 as to $\Delta E_{6.5\,\mathrm{T}}$ so they have no effect on the difference Δ, and can be neglected. For the Zeeman energy of J = ½ ground state, we use the directly measured "m_J-flip" excitation energy, which for Sm-bv at B = 6.5 T is $g_{1/2}\mu_B B/2$ = 0.9465 mV (Figure 4c). Then we need to compute only the eigenstates of the J = 3/2 manifold and use the resulting energy eigenvalues to compute the excitation energies that we match to the experiment. The Hamiltonian for the J = 3/2 multiplet is thus simplified to

$$H_{3/2} = g_{3/2}\mu_B \boldsymbol{J}\cdot\boldsymbol{B} + DJ_z^2 \qquad \text{(Eq. S4)}$$

Here $g_{3/2}$ is the effective g-factor of the J = 3/2 multiplet, which arises from the unknown g-tensor and our magnetic field direction. This simplified Hamiltonian is intended to capture the main effects of a finite axial magnetic anisotropy term D that tends to orient the spin towards or away from a specific direction. The shift is given by

$$\Delta = E_{3/2}(B = 6.5\,T) - E_{3/2}(B = 0) + g_{1/2}\mu_B B/2 \qquad \text{(Eq. S5)}$$

Here $E_{3/2}$ are the energy eigenvalues obtained from the simplified Hamiltonian (Eq. S4) evaluated at either B = 0 T or 6.5 T as shown. We then obtain $g_{3/2}$ and D numerically as the desired fitting parameters. We find that for D oriented along any of the three crystal symmetry directions (normal to the surface plane, along the O-O bridge direction or along the Mg-Mg bridge direction), and with either sign of D with magnitude ranging from 0.20 mV to 10 mV, the result gives a g-factor $g_{3/2}$ is less than 1, an implausibly small value, for nearly all cases considered. There are two exceptions, which both require D < 0 oriented normal to the surface plane (corresponding to easy-axis anisotropy) (Figure S13d), so that the external field is oriented mostly orthogonal to the anisotropy axis in our measurement. The first is the situation in which D ranges from −0.200 to −0.275 mV, for which $g_{3/2}$ ranges from 1.95 to 2.1, corresponding to an excitation $\left|\frac{1}{2}, -\frac{1}{2}\right\rangle \rightarrow \left|\frac{3}{2}, -\frac{1}{2}\right\rangle$ with $\Delta m_J = 0$. The second is the situation in which D ranges from −1 to −2 mV, for

which $g_{3/2}$ ranges from 1.6 to 2.0, corresponding to an excitation $\left|\frac{1}{2},-\frac{1}{2}\right\rangle\rightarrow\left|\frac{3}{2},-\frac{3}{2}\right\rangle$ with $\Delta m_J = -1$. Since only one excitation is observed, both cases imply a selection rule given by the stated Δm_J, whose justification is beyond the scope of this work to evaluate theoretically, and we consider both cases as plausible explanations of the measurements.

Consideration of IETS cross sections. Examination of the IETS cross sections (step heights in d*I*/d*V*) for Sm-b and Eu-b is revealing. For Eu-b, there are two transitions observed in IETS: the m_J -changing transition $|4,-4\rangle\rightarrow|4,-3\rangle$, which occurs at the Zeeman energy of ~0.74 mV at 6.5 T with conductance change Δσ/σ ≈ 17%; and the 6*s*-flip transition $|4,-4\rangle\rightarrow|3,-3\rangle$, which occurs at ~163 mV and is much more prominent, with Δσ/σ ≈ 187% (Figure 1 and Figure S2). Here σ is the elastic conductance, given by the conductance at voltages below the first IETS step. The high efficiency of the 6*s*-flip can be attributed qualitatively to the ease with which the 6*s* electron alone can be flipped by tunneling electrons. It flips this 6*s* spin against the entire 4*f* electron manifold at the expense of an intra-atomic exchange energy penalty[28]. In contrast, the m_J-changing transition requires changing m_J for the joint wavefunction of entire 6*s*-4*f* coupled spin system. Assuming that tunneling electrons interact directly only with the 6*s* orbital during the inelastic scattering process, as the 4*f* orbital is shielded from the tunneling electrons, it is reasonable that 6*s*-flip transitions should be much more efficient than magnetic transitions that involve changes to the *f*-shell.

Examining the comparable IETS cross sections for Sm-b, the m_J-changing transition, which occurs at the Zeeman energy of ~1.8 mV at 6.5 T, has cross section Δσ/σ ≈ 10.5 %. The 6*s*-flip transition (at ~148 mV) has IETS cross section Δσ/σ ≈ 140% (Figure 4). That the IETS cross sections are lower for Sm-b than for Eu-b may be due to the nonzero *L* of Sm-b, which imparts additional magnetic degrees of freedom for the *f*-shell electrons involved in the transition that are isolated from the tunneling electrons. The *L*-*S*-tilt transition for Sm-b (at ~38 mV) has cross section Δσ/σ = 56%, but there exists no comparable excitation for Eu. Note that the somewhat larger IETS cross section for Eu than for Sm for the 6*s*-flip excitation was also observed previously for Sm and Eu adsorbed on metal-supported graphene[28]. For each element, we observe a ratio between the 6*s*-

flip and m_J-changing transitions of ~11–13, which confirms that these transitions are of similar character.

Summary. This section considered alternative interpretations of the *L*-*S*-flip excitation at ~38 meV and we conclude that these measurements are consistent with either $\left|\frac{1}{2}, -\frac{1}{2}\right\rangle \rightarrow \left|\frac{3}{2}, -\frac{1}{2}\right\rangle$ and *D* = 0 to –0.275 meV, or alternatively $\left|\frac{1}{2}, -\frac{1}{2}\right\rangle \rightarrow \left|\frac{3}{2}, -\frac{3}{2}\right\rangle$ with *D* = – 1 to –2 meV, in both cases with *D* oriented normal to the surface plane.

6. State assignments for the Sm-O IETS transitions

For Sm on the oxygen binding site, Sm-O, we observed excitations at ~26 mV and ~89 mV (Figure S12) that we attribute to *L*-*S*-tilt transitions, which are excitations into the *J* = 3/2 multiplet ($^8F_{3/2}$). As for Sm-b, we expect that the crystal field will split this multiplet into two doublets, which have m_J = ±1/2 and m_J = ±3/2. The diagrams in Figure S13c–e provide candidate energy orderings. Our spectroscopic data do not include sufficient detail to assign a specific doublet to each excitation energy, but we comment on the possibilities below.

Due to the difficulty of studying this species (for most tip apexes, we were not able to easily reposition Sm to the O-site), we do not have data at elevated bias voltages (>100 mV) as we do for Sm-b. At elevated bias ranges both Sm-b and Sm-O readily hop to the Mg-site, requiring the tip to be backed out increasingly far from the surface as the bias range is elevated to avoid this scenario, which in turn reduces signal/noise. Accordingly in Figure S12 we obtained tunneling spectra for Sm-O only up to 100 mV, which is not high enough to approach the 6*s*-flip transition energy ~148 mV observed for Sm-b or 188 mV for gas-phase Sm^+. Nevertheless, we observe an IETS transition at ~89 mV. Following the above arguments that 6*s*-flip transitions should have the greatest IETS intensity among observable transitions, we assign the ~89 mV excitation, which is weaker than the ~26 mV excitation, to an *L*-*S*-tilt excitation $J = \frac{1}{2} \rightarrow J = \frac{3}{2}$. In this case the higher energy arises from zero-field splitting of the $J = \frac{3}{2}$ multiplet rather than to the 6*s*-flip transition. We point out that in the regime where the magnetic anisotropy energy becomes comparable to the

spin-orbit coupling, J and m_J are not good quantum numbers due to state mixing, so that Figure S13e is oversimplified.

In the discussion for Sm-b above, we could not discern the size of the splitting of the $J = \frac{3}{2}$ states into two doublets, since selection rules appear to prohibit transitions to some of the available excited states. As selection rules can be weakened or modified by the crystal field, and the crystal field environment of Sm-O may differ considerably from that of Sm-b, it is plausible that Sm-O may have different selection rules governing which $J = \frac{1}{2} \rightarrow J = \frac{3}{2}$ transitions are allowed in IETS. We assign the Sm-O spectrum to the excitation scheme shown in Figure S13e. Due to the C4v symmetry of the oxygen-atop binding site, D is expected to be oriented normal to the surface plane and $E = 0$. Applying a similar analysis using Eq. S3 and S4 as was done for Sm-b, we obtain $|D|$ = 31 mV from the splitting of the 26 mV and 89 mV excitations, but cannot determine the sign of D since the expected Zeeman splitting for the J = 3/2 state is too small with the external field B oriented nearly orthogonal to D. Evidently the entire shift of the ~26 mV excitation at B = 6 T seen in Figure S12 arises from the lowered ground state energy of the $\left| \frac{1}{2}, -\frac{1}{2} \right\rangle$ state, rather than any magnetic field dependence of any of the J = 3/2 states.

Alternatively, while the ~26 mV feature is due to a $J = \frac{1}{2} \rightarrow J = \frac{3}{2}$ L-S-tilt excitation, the ~88 mV feature could instead correspond to the $J = \frac{1}{2} \rightarrow J = \frac{5}{2}$ L-S-tilt excitation, which is normally forbidden because $\Delta J = 2$. The $J = \frac{5}{2}$ state is 103.9 mV above the ground state for the gas phase Sm^+ ion[35].

Future studies which employ a B field oriented normal to the surface plane (and parallel to D) could conclusively determine the correspondence of the 26 mV and 89 mV excitations to the J = 3/2 or J = 5/2 states.

7. Origin of large Landé *g*-factors for Sm

This section describes how large Landé g-factors arise, in order to explain the large, measured g-factor (~4.9) for Sm on the bridge site of the MgO film (Sm-b). In most atoms and ions, the Landé g-factor falls between the electron orbital g-factor $g_{\mathrm{L}} = 1$ and the electron spin g-factor $g_{\mathrm{e}} = 2.0023$. Examination of the formula for the Landé g-factor shows that large g-factors arise in atoms and ions for which: (1) S and L are both large, (2) S slightly exceeds L, and (3) the valence shell is less than half full so that Hund's third rule applies to give a value of $J = S - L$ that is small but non-zero. These conditions apply in the case of the ground state of free Sm^{+} to give $g_J = 4$. Under the approximation that the electron g-factor is $g_e \approx 2$, an atom or ion's Landé g-factor is given by

$$g_J = \frac{3}{2} + \frac{S(S+1) - L(L+1)}{2J(J+1)}$$

For $S > L$, and when J is minimized so that $J = S - L$, we rewrite the g-factor in terms of L and J as

$$g_J = \frac{3}{2} + \frac{(L+J)(L+J+1) - L(L+1)}{2J(J+1)}$$

$$= 2 + \frac{L}{J+1}$$

The plot shown in Figure S13a illustrates this behavior. This expression shows that minimizing J for a given L maximizes the g-factor. For the smallest positive half-integer value, $J = ½$,

$$g_J = 2 + \frac{2}{3}L$$

For gas-phase Sm^{+}, we have $L = 3$ and $g_J = 4$, which is the largest known g-factor value for any free atom or ion in its ground state. This limit arises from observing that exceeding $L = 3$ and simultaneously having $S \geq L$ is a condition that would require S to be at least 9/2. This large S might be plausible by having the f, s, and d subshells all incompletely filled, such as in the hypothetical $4f^6 5d^2 6s^1$ configuration, where the spin is maximized to give $S = 9/2$, and the orbital angular momentum is selected to give $L = 4$. Such a term is not known to exist in any free ion's ground state, but it would theoretically yield the large value $g_J = \frac{14}{3}$.

The g-factors we observe for Sm-b lie in the range 4.71–5.04, which significantly exceeds even the anticipated limit of 4. The analysis above of the Landé g-factor applies when J is a good quantum number, to useful approximation. We expect that the stationary quantum states of atoms in an asymmetric environment (such as adsorbed on surface) will be superpositions of J states. We propose that the large g-factor of Sm-b arises from partial transfer of the 6s electron to the 5d subshell where it can exhibit orbital angular momentum that increases the magnetic moment of the two observed quantum states that are loosely characterized by $m_J = \pm \frac{1}{2}$.

The most extreme negative g-factors may be obtained in cases where S and L nearly cancel but where L exceeds S. Minimizing J yields g-factors of largest magnitude. Letting $J = L - S$,

$$g_J = \frac{3}{2} + \frac{(L-J)(L-J+1) - L(L+1)}{2J(J+1)}$$

$$= 2 - \frac{L+1}{J+1}$$

This expression gives a g-factor of −2/3, when $L = 3$ as for gas-phase Sm^+. In this case $J = 1/2$ and $S = 5/2$ (term $^6F_{1/2}$). We note that the $^6F_{1/2}$ excited state of gas-phase Sm^+ (which has a $^8F_{1/2}$ ground state) has a known energy of 188 mV and g-factor of −0.595, which is close to this predicted extreme. We observe this excitation at ~148 mV for Sm-b.

8. Stabilizing Ln(I) on surfaces

In the main text we proposed that adsorption on metal-supported thin insulating films may serve as a general route to stabilizing open-valence-shell lanthanides in the solid state. Here we discuss this idea further. In previous studies of lanthanides adsorbed on 2 ML MgO grown on Ag(001), individual atoms such as Ho, Dy and Er underwent charge transfer to the underlying substrate to form a +1 cation[3,24,28], as did Eu and Sm in the present work. A key property in determining whether a given element shows an open valence shell cation is its propensity to form a divalent configuration in compounds, as in Eu and Sm, versus a trivalent configuration, as in Ho, Dy and

Er, which instead promote a 4*f* electron to the 5*d* shell. The combined result of this 4*f*-5*d* electron promotion and cation formation is a closed-shell $6s^2$ valence configuration, with the 5*d* electron having transferred to the underlying substrate. For Eu and Sm, the stronger preference for a divalent configuration, and the consequent lack of 4*f*-5*d* electron promotion, means that cation formation instead results in the desired $6s^1$ open-shell valence configuration, which we propose is the key to electronic access to their magnetic properties.

We note here several aspects that stimulate further investigation:

1) We expect that other lanthanides with a preference for a divalent electron configuration, such as Yb and Tm[30], should also form monovalent cations on 2 ML MgO/Ag(001) similar to Eu and Sm.

2) As noted in refs.[51–53], ultra-thin insulating films (< 5 ML) modify the work function of the surface, facilitating charge transfer to or from adsorbates. Different combinations of insulating film material, thickness, and underlying conductor influence the propensity of adsorbates to undergo charge transfer, so they offer a rich state space to explore for studies of open-shell Ln(I).

3) Most lanthanides studied in the graphene-on-metal adsorption environment showed easily detected magnetic excitations in IETS, so they were also likely monovalent[28]. In experiments on borozene clusters, lanthanides having a divalent preference such as Tm and Yb, in addition to those with stronger trivalent preference (La, Pr, but notably not Tb) could similarly be stabilized in $6s^1$ monovalent configurations[27]. These observations suggest that details of the local adsorption environment influence whether individual Ln atoms promote 4*f* electrons to the 5*d* shell, in addition to their element-specific propensities. We can expect that other thin insulating films aside from MgO may result in a greater or lesser propensity for adsorbed lanthanides to undergo 4*f*-5*d* promotion. These other thin films may allow elements other than Eu, Yb, Sm and Tm to be stabilized in the $6s^1$ monovalent configuration, including some of the more trivalent-preferring lanthanides.

The above considerations warrant further theoretical and experimental investigation in understanding what factors influence 4*f*-5*d* electron promotion in a surface adsorption environment, which is distinct from most other chemical environments due to the under-coordination of the Ln ion. As we have seen in the present work, there are also variations in charge or valence configuration which depend on the particular adsorption site on a given substrate, exemplified by the change to a closed-shell configuration when Eu and Sm are adsorbed on the Mg-site. We anticipate that a better developed understanding of these factors will lead to more studies that take advantage of the unique chemical and magnetic properties of monovalent lanthanides.

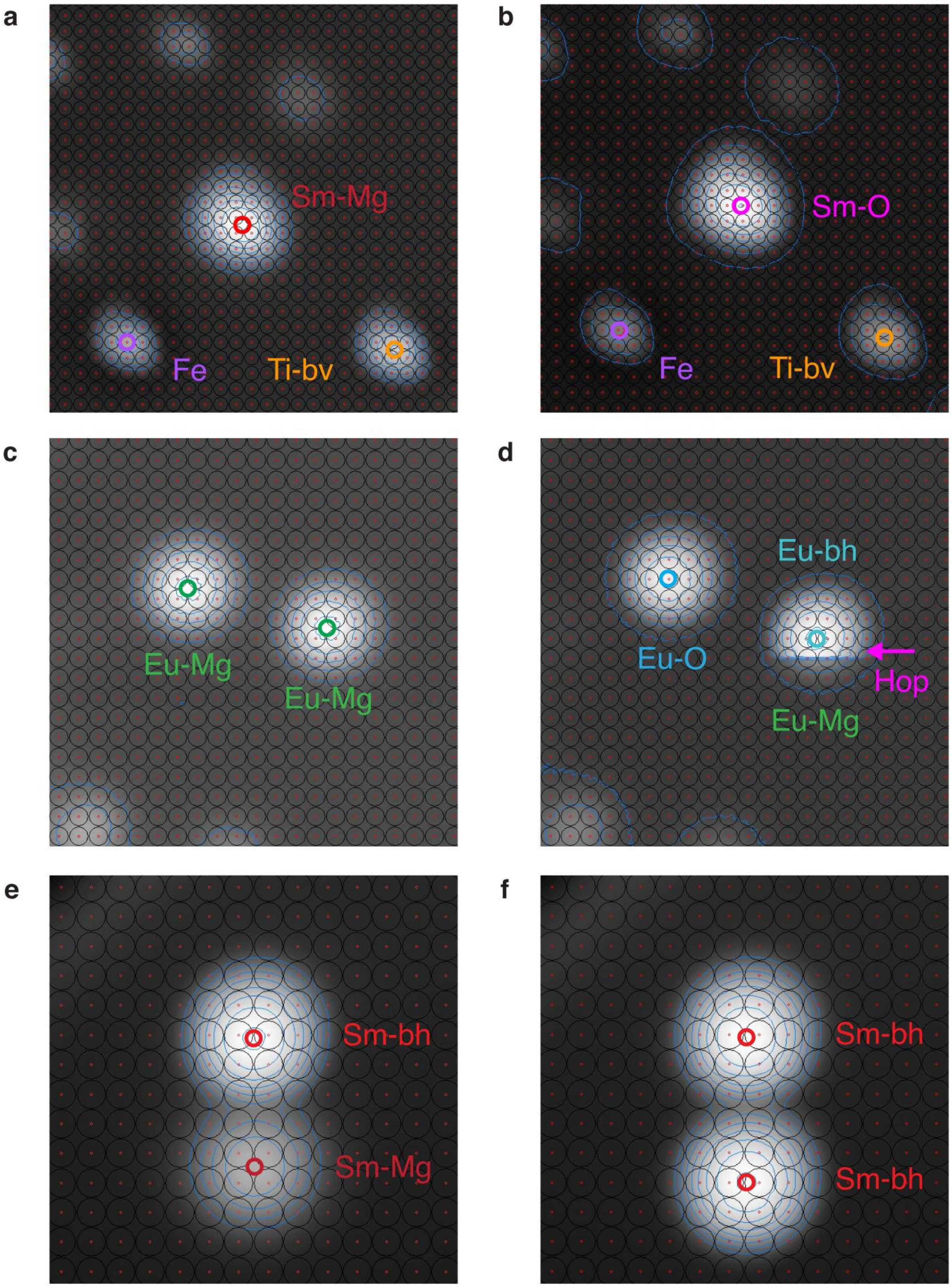
a
Sm-Mg
Fe
Ti-bv
b
Sm-O
Fe
Ti-bv
c
Eu-Mg
Eu-Mg
d
Eu-bh
Eu-O
Hop
Eu-Mg
e
Sm-bh
Sm-Mg
f
Sm-bh
Sm-bh

Figure S1. STM images and binding sites for Eu and Sm on MgO film.

(**a**) Binding site of Mg-site Sm (Sm-Mg) depicted with underlying MgO lattice (red points represent oxygen atoms). Nearby oxygen-site Fe (Fe-O) and vertical-bridge-site Ti (Ti-bv) atoms are used to align the lattice. (**b**) Binding site of Sm-O after using atom manipulation to reposition the Sm atom in (a). (a) and (b) were acquired with current setpoint I_{set} = 15 pA at V_{set} = 50 mV, image size 7.5 nm square. (**c**) Binding site of Eu-Mg atoms with lattice overlay. (**d**) Binding sites of Eu-O and horizontal-bridge-site Eu (Eu-bh) after using atom manipulation to reposition the rightmost Eu-Mg to the bh site. Unusually, this tip apex would concurrently reposition the neighboring Eu-Mg atom (left) to the O-site when performing atom manipulation with the tip apex centered on the rightmost Eu. The rightmost Eu atom can be seen to hop at the labeled scan line from the bh site to the Mg site during image acquisition. (c) and (d) acquired with 5 pA at 50 mV, image size 6 nm square. (**e**, **f**) Binding sites of Sm-bh and Sm-Mg before (e) and after (f) atom manipulation used to reposition the bottom Sm atom to the bh site. (e) and (f) acquired with 10 pA at 50 mV, image size 4 nm square.

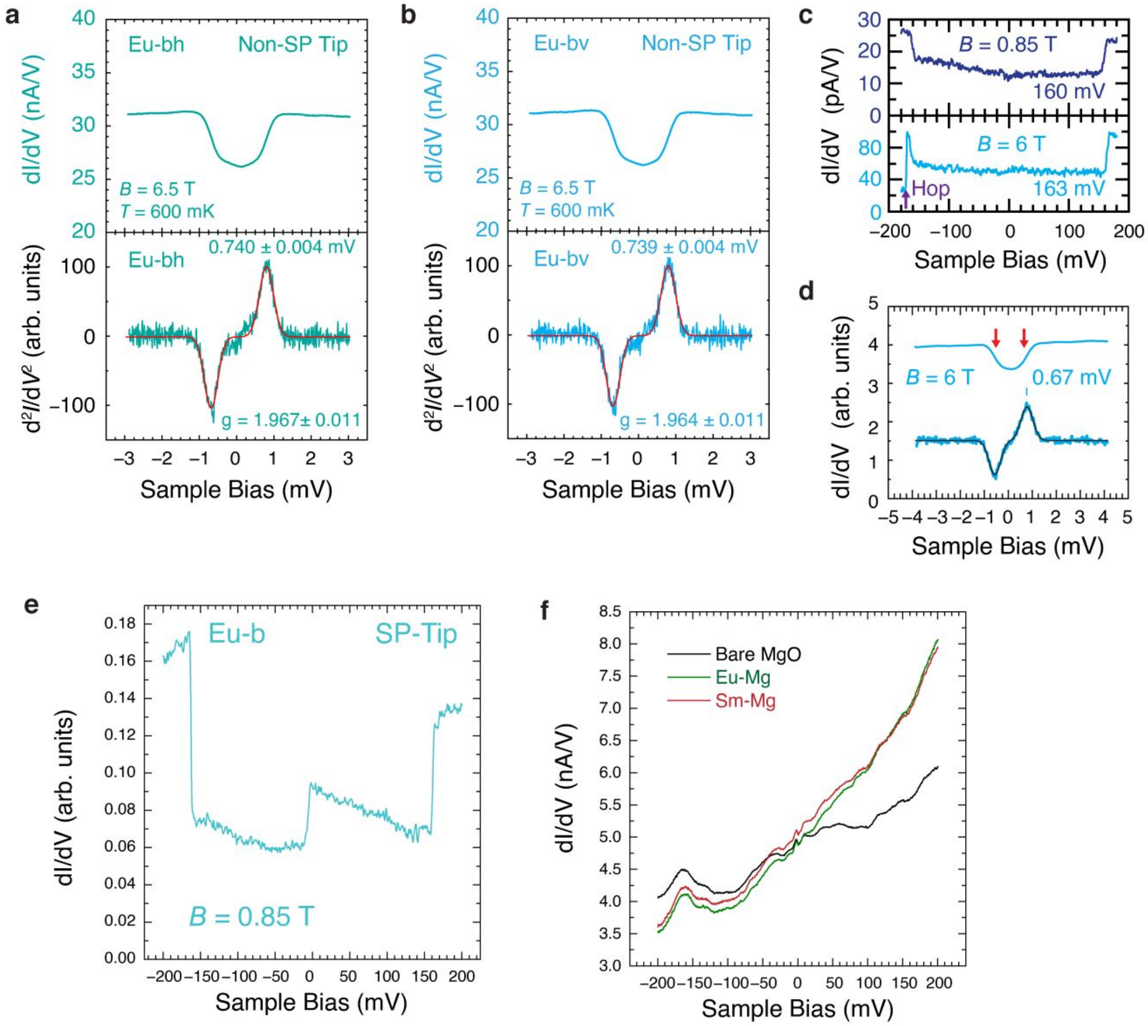

Figure S2. Additional tunneling spectra of bridge-site Eu (Eu-b).

(**a**) Tunneling spectra of horizontal bridge-site Eu (Eu-bh), acquired at tip-height setpoint 300 pA at 10 mV, AC modulation $V_{AC} = 100$ µV, $B = 6.5$ T, $T = 600$ mK. (**b)** Tunneling spectra of vertical bridge-site Eu (Eu-bv), same conditions as (a), reproduced from main text Figure 1f. In (a) and (b), a Gaussian fit to each peak, averaged over positive and negative bias, gives energy as labeled. Uncertainties in *g*-factors shown in the figure are the result of peak-fitting uncertainty, and do not include the 0.5% uncertainty in the applied magnetic field. No difference in the excitation energy is discerned between the two orientations, bh and bv, providing evidence that Eu-b has negligible in-plane magnetic anisotropy and high in-plane symmetry of the *g*-tensor. (**c)** Tunneling spectra of Eu-b acquired using two additional tip apexes at 0.85 T (top) and 6 T (bottom). Each spectrum was acquired with a unique tip apex which differed from the tip apex used in Figure 1e of the main text. Top: tip-height setpoint $I_{set} = 2.5$ pA at $V_{set} = 80$ mV. Bottom: setpoint 5 pA at 80 mV. For both spectra, $V_{AC} = 3$ mV rms, $T = 600$ mK. (**d)** Low bias tunneling spectra of Eu-b showing the Zeeman splitting at 6 T, acquired with feedback setpoint 200 pA at 10 mV, $V_{AC} = 200$ µV, $T = 600$ mK. Top trace: dI/dV; bottom trace: dI^2/dV^2 numerically computed. (**e**) Tunneling spectra of Eu-b acquired with a spin-polarized tip, setpoint 40 pA at 20 mV. Note the abrupt conductance step near zero bias and the large conductance step height asymmetry between positive and negative bias for

the 163 mV excitation. These asymmetries arise from spin-polarized IETS selection rules and further evidence the magnetic nature of both the zero-bias excitation and the 163 mV excitation of Eu-b[34]. (**f**) Tunneling spectra of Eu-Mg, Sm-Mg and the bare MgO substrate as a background measurement. No IETS features were observed for either Eu-Mg or Sm-Mg, in contrast to b-site and O-site adsorbed species. All spectra in (f) were acquired with tip-height setpoint 50 pA at 10 mV, V_{AC} = 1 mV, B = 0 T.

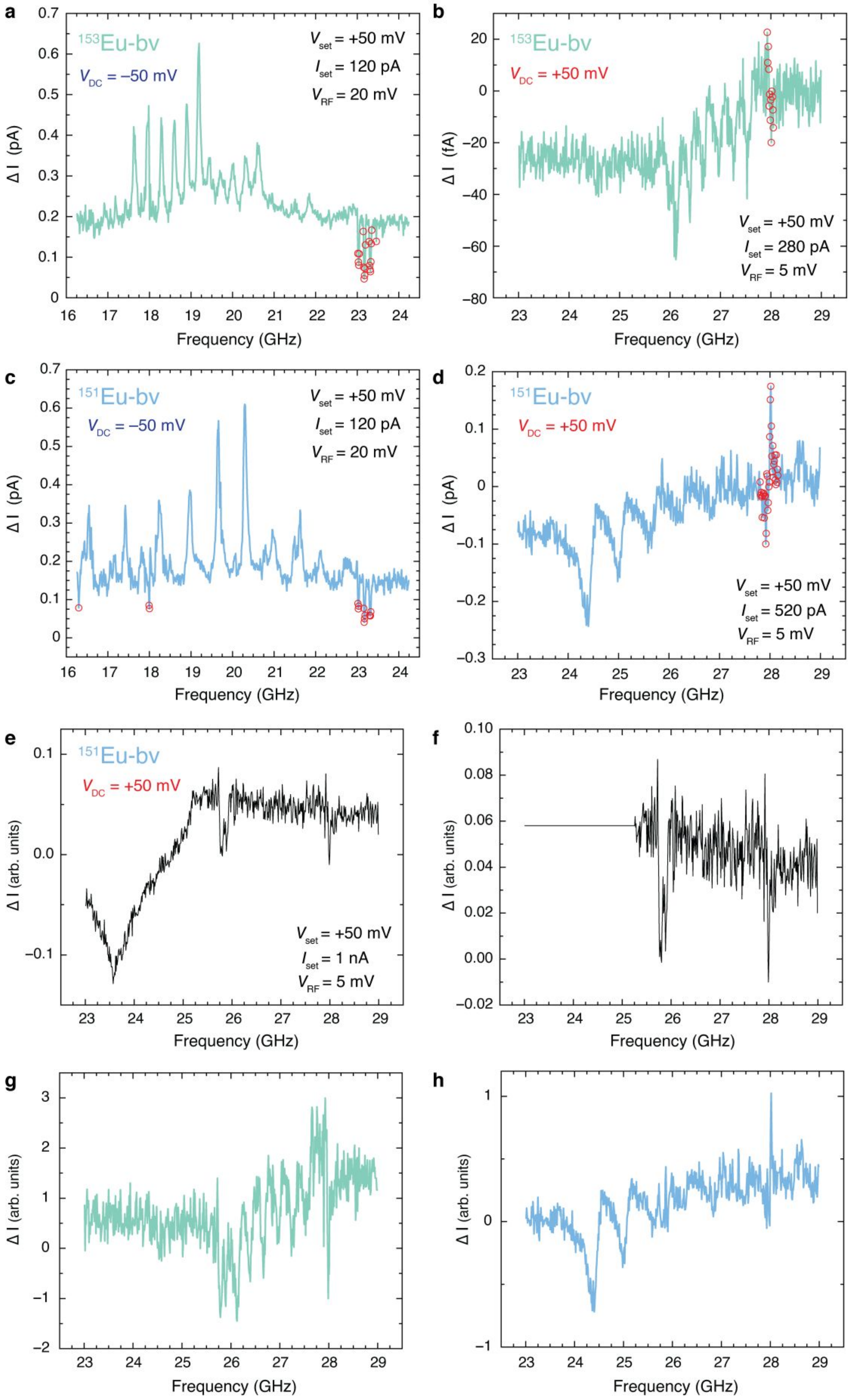

a
153Eu-bv
VDC = –50 mV
Vset = +50 mV
Iset = 120 pA
VRF = 20 mV
Δ I (pA)
Frequency (GHz)
b
153Eu-bv
VDC = +50 mV
Vset = +50 mV
Iset = 280 pA
VRF = 5 mV
Δ I (fA)
Frequency (GHz)
c
151Eu-bv
VDC = –50 mV
Vset = +50 mV
Iset = 120 pA
VRF = 20 mV
Δ I (pA)
Frequency (GHz)
d
151Eu-bv
VDC = +50 mV
Vset = +50 mV
Iset = 520 pA
VRF = 5 mV
Δ I (pA)
Frequency (GHz)
e
151Eu-bv
VDC = +50 mV
Vset = +50 mV
Iset = 1 nA
VRF = 5 mV
Δ I (arb. units)
Frequency (GHz)
f
Δ I (arb. units)
Frequency (GHz)
g
Δ I (arb. units)
Frequency (GHz)
h
Δ I (arb. units)
Frequency (GHz)

Figure S3. Raw data for ESR spectra of Eu-b shown in main text Figure 2.

(**a**, **b**) ESR spectra for ^{153}Eu. (**c**, **d**) ESR spectra for ^{151}Eu. For (a–d), the spectra are the same data as those shown in the main text Figure 2, here plotted without smoothing. Here the entire spectrum is shown including points dominated by artifacts that arise from our instrument's uncompensated frequency-dependent transfer function at certain frequencies (marked in red). The red points were omitted in the main text for clarity. (**e**) ESR spectrum for ^{153}Eu acquired at tip-height setpoint I_{set} = 1 nA at I_{set} = 50 mV, V_{RF} = 5 mV 0-p (zero-to-peak). The closer tip proximity redshifted the ESR features toward 24 GHz, so that the 25.5–29.5 GHz frequency region was free of ESR signals so it could be used as a background measurement to subtract artifacts due to the uncompensated transfer function. (**f**) Same as (e) but with the frequency range containing ESR signals (< 25.5 GHz) removed and replaced with a constant background. The spectrum in (f) was subtracted from the raw data to obtain the main text Figure 2 spectra acquired at positive bias, and to obtain spectra in (b) and (d) shown here. (**g**, **h**) Raw data for ^{153}Eu and ^{151}Eu, respectively, before any background subtraction.

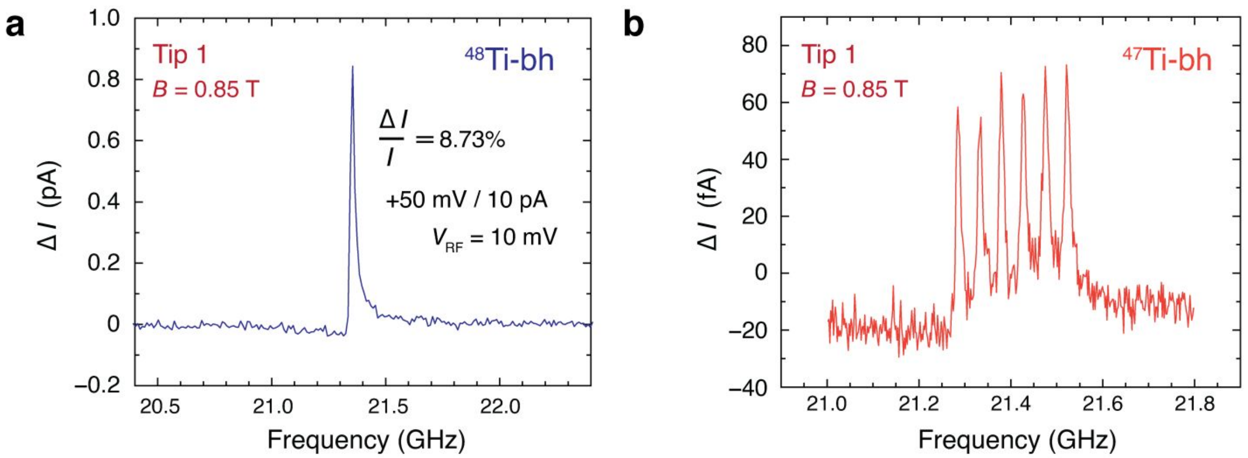

Figure S4. ESR spectra of Ti isotopes ^{48}Ti-b and ^{49}Ti-b.

(**a**) ESR spectrum of ^{48}Ti-b (nuclear spin $I = 0$) at $B = 0.85$ T. The strong signal/noise of the apex used to acquire this data resulted in an ESR peak amplitude of over 800 fA, which represents a change in tunneling current of ~8.7 % on resonance. (**b**) ESR spectrum of ^{47}Ti-b ($I = 5/2$, giving 6 peaks) acquired with the same tip apex under similar conditions, averaged over 4 passes. The peak heights are less than one sixth of those for the zero nuclear spin isotope because each peak corresponds to one of the six thermally occupied nuclear spin states. (a) and (b) use "Tip 1", the same tip apex used to acquire all ESR data shown in the main text Figures 2, 3 and 5, and supplementary Figures S3, S4, S6, S7, S14d–g, and S15. Since the Eu ESR signal intensity of each peak is significantly reduced owing to the large number of electron and nuclear spin states, the quality and signal-to-noise ratio for magnetic tips needs to be especially high for studying Eu. We provide the Ti-b spectra here as a reference for this tip's performance.

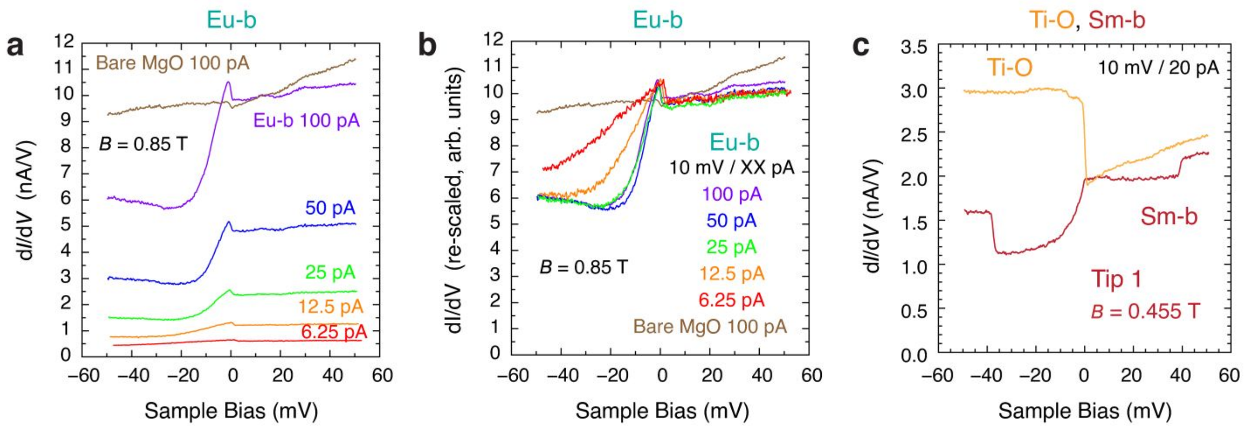

Figure S5. Spin torque of Eu-b and detection of tip spin polarization using Ti-O.

(**a**) Tunneling spectra (dI/dV) of Eu-b at varying tip-surface distance, established by varying the tip-height setpoint current as indicated with each trace, at V_{set} = 10 mV, V_{mod} = 0.5 mV rms, B = 0.85 T. All traces shown were acquired with the spin-polarized tip positioned over Eu-b, except the brown trace which was acquired with the tip positioned over the bare MgO film as a background measurement at a position laterally far from any adsorbate. (**b**) Data shown in (a) with each trace re-scaled vertically by normalizing to the I = 100 pA trace based on the ratio of setpoint currents. Negative-bias dropoff of conductance as current is increased indicates spin torque[38]. The saturation of the magnetoconductance (conductance drops half-way from the low-voltage value to the high-voltage limiting value) occurs at ~20 pA, or one tunneling electron per ~8 ns. Estimating from the IETS step height (Fig. 1f) that ~15% of the tunneling electrons relax the atomic spin, we estimate the intrinsic spin relaxation time $T_1 \approx 50$ ns. (**c**) Tunneling spectra of Sm-b and Ti-O acquired with the same tip apex at setpoint 20 pA at 10 mV, B = 0.455 T, T = 0.6 K. The conductance change near zero bias for Ti-O arises from spin-polarized inelastic excitation selection rules and can be used to characterize the spin polarization of the tip. The tip used here, “Tip 1”, was the same tip apex used to acquire all ESR data in main text: Figures 2, 3 and 5, and supplementary Figures S3, S4, S6, S7, S14d–g and S15.

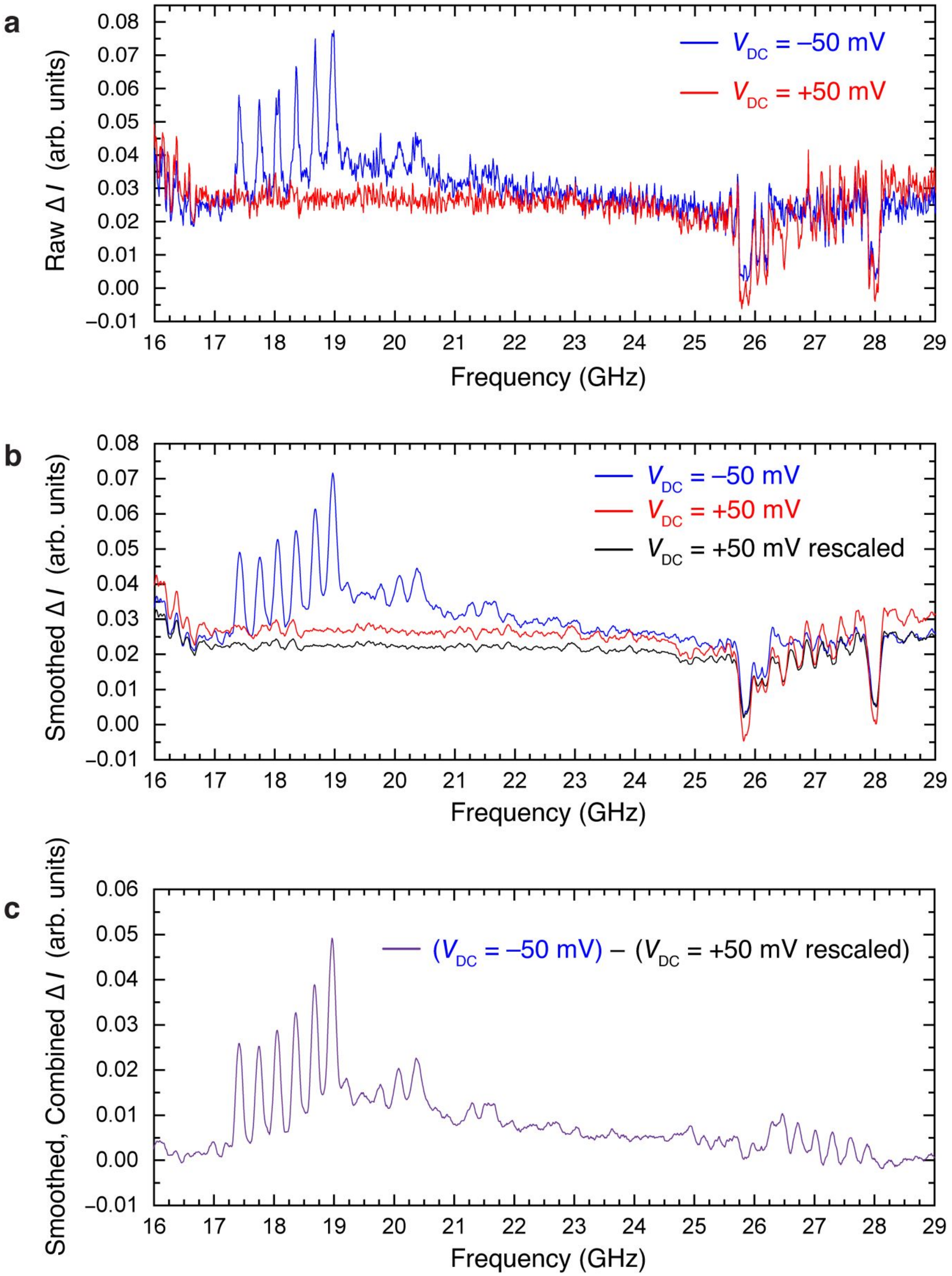
a
Raw Δ I (arb. units)
V_DC = –50 mV
V_DC = +50 mV
Frequency (GHz)
b
Smoothed Δ I (arb. units)
V_DC = –50 mV
V_DC = +50 mV
V_DC = +50 mV rescaled
Frequency (GHz)
c
Smoothed, Combined Δ I (arb. units)
(V_DC = –50 mV) – (V_DC = +50 mV rescaled)
Frequency (GHz)

Figure S6. Processing ESR spectra for ^{153}Eu-b shown in main text Figure 3.

(**a**) ESR spectra acquired with the tip positioned over a ^{153}Eu-bv atom (bridge-site in vertical direction) with tip-height setpoint I_{set} = 67 pA at V_{set} = −50 mV (blue curve) and I_{set} = 200 pA at V_{set} = +50 mV (red curve); B = 0.85 T, V_{RF} = 10 mV. These two setpoints are approximately identical in tip height (tip-surface distance) despite the difference in tunnel junction conductance because of the larger magneto-conductance at positive bias found for this junction. (**b**) Positive and negative bias spectra from (a) smoothed using the Savitzky-Golay method with a 20-point averaging window. The black curve corresponds to re-scaling the red (positive bias) curve by a factor 0.65 to approximately match the signal strength of the rectification background and transfer-function anomalies present in the negative bias (blue) curve. (**c**) Subtracted, smoothed data, also shown as Figure 3a of the main text. The rescaled, smoothed positive bias data (black curve in (b)) was subtracted from the smoothed negative bias data (blue curve in (b)) to produce the complete spectrum shown in purple. Since the ESR signal at positive and negative bias had opposite amplitude, subtracting the positive bias data (where ESR signals are dips) from the negative bias data (where ESR signals are peaks) produces a spectrum that shows ESR features from both bias polarities as peaks while largely cancelling the transfer function artifacts present in both original spectra. This method allows a more complete view of the ^{153}Eu ESR spectrum, by combining the data resulting from spin-torquing the electron spin toward the ground state (positive bias) and spin-torquing toward the most excited states (negative bias).

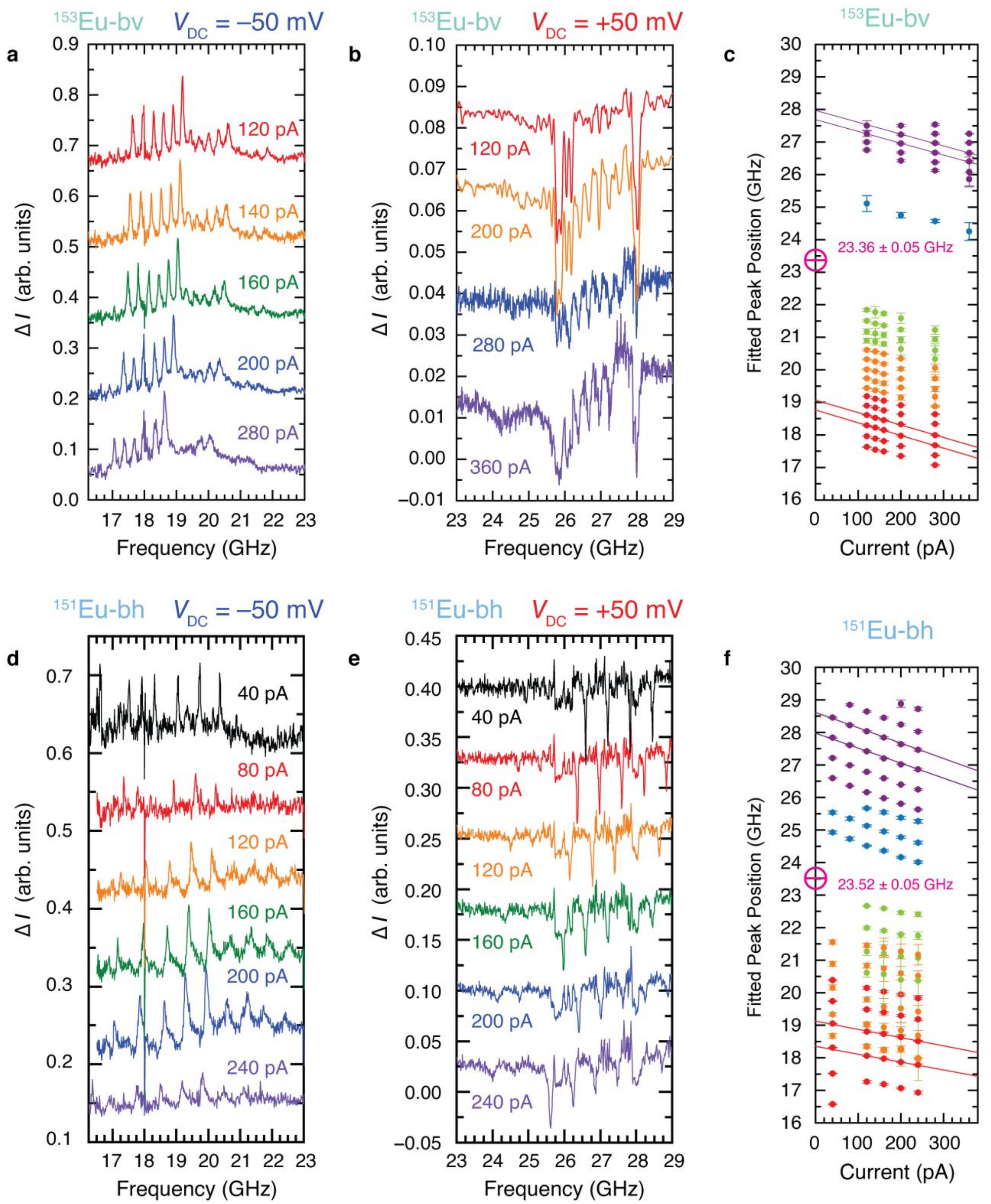

Figure S7. Determining the *g*-factor of Eu-b from ESR spectra.

(**a**) ESR spectra for ^{153}Eu-bv acquired at different tip heights, established by varying the setpoint tunneling current as labeled at $V_{set} = +50$ mV. Bias voltage during the ESR acquisition was $V_{DC} = -50$ mV. (**b**) ESR spectra acquired at same conditions except $V_{DC} = +50$ mV. (**c**) Fitted peak positions for all spectra shown in (a) and (b). Linear fits to the peak positions for tree 1 peaks 3 and 4 (red lines) and tree 8 peaks 3 and 4 (purple lines) are shown. The y-intercepts of these four linear fits were then averaged to approximate the Zeeman energy (pink circle on vertical axis) of the atom with the effects of the crystal field and tip magnetic field mostly cancelled. (**d**–**f**) Similar

ESR spectra and resulting fitted peak positions for a different isotope and bridge orientation, ^{151}Eu-bh. For all panels, $B = 0.85$ T and $V_{RF} = 20$ mV for negative bias spectra, $V_{RF} = 10$ mV for positive bias spectra. All spectra were acquired with feedback loop open after setting the tip height using the indicated setpoint currents at $V_{set} = +50$ mV. For negative bias spectra the feedback was established at positive bias and then the bias was changed to $V_{DC} = -50$ mV under open loop conditions before data acquisition. (An exception was the 40 pA spectrum for $V < 0$ in which the bias was instead ramped to $V_{DC} = -100$ mV to improve signal strength; this data point was not included in the linear fits). The extrapolated Zeeman energy provides an estimate of the electron g-factor, giving 1.964 ± 0.004 for ^{153}Eu-bv and 1.977 ± 0.004 for ^{151}Eu-bh. These error bars arise from the errors in the peak fits, but do not take into account uncertainties in the total magnetic field, which includes the applied magnetic field (0.5% uncertainty) and the unknown tip dipole magnetic field. This tip dipole field is uncharacterized and can be aligned along or orthogonal to the in-plane transverse anisotropy E direction, which may account for the difference in apparent g-factors between the two measurements shown. See Supplementary Note 2 for further discussion. Spectral features near 18 GHz, 25.75 GHz and 28 GHz are artifacts due to the microscope's uncompensated frequency-dependent RF transfer function.

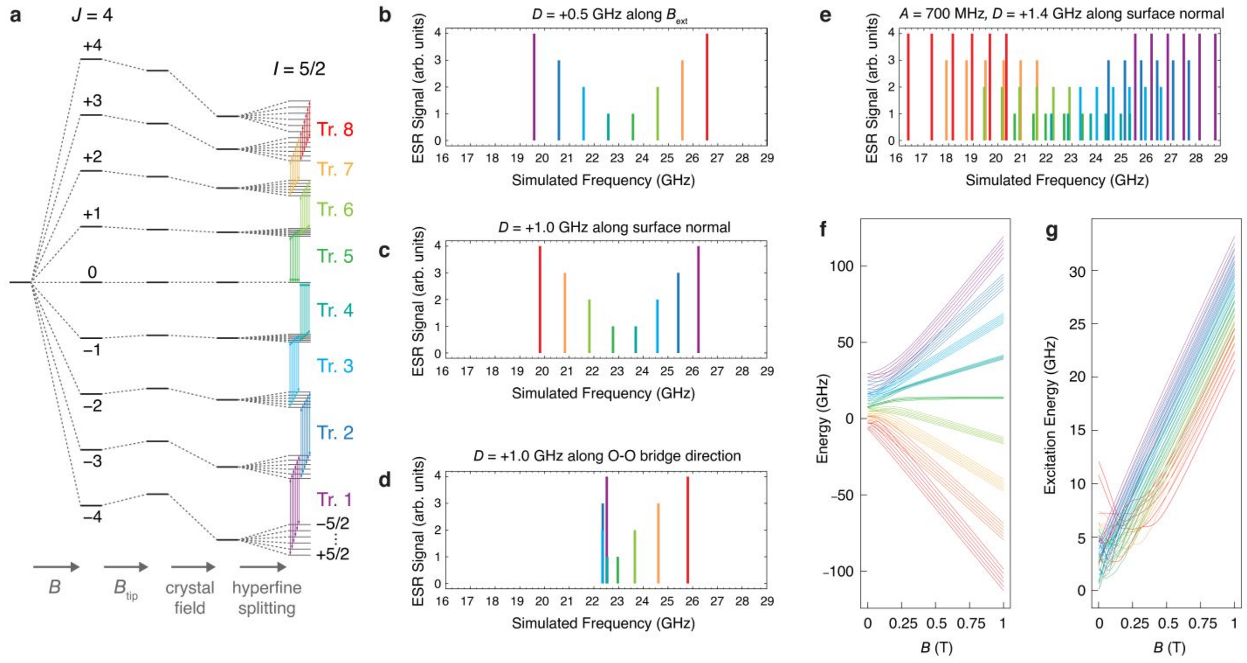

Figure S8. Calculated states and transitions from model Hamiltonian of Eu-b.

(**a**) Schematic energy level diagram showing the qualitative effect of each term of the model Hamiltonian, for the $J = 4$, $I = 5/2$ spin system of Eu^{+}. The effects of each term are not drawn to scale. (**b**) Simulated excitation energies assuming an axial magnetic anisotropy term $D = +0.5$ GHz oriented along the external magnetic field with $B = 0.85$ T. For illustration, hyperfine interactions are excluded here, so that there are 9 magnetic states (8 electron spin-flip transitions). The depicted height of each excitation is arbitrary and chosen to be suggestive of heights seen in measured spectra. Colors depict the ordering of the initial energy eigenstate, with red corresponding to excitations between the ground state and first excited state, and purple corresponding to excitations between the second-most and most excited energy state. (**c**) Simulated excitation energies for $D = +1.0$ GHz oriented normal to the surface plane, assuming $B = 0.85$ T oriented mostly in-plane with a 9° tilt toward the surface normal. Note the reversed excitation energy ordering relative to (a) that results. The energy spacing between excitations is no longer equal, with a slightly larger spacing occurring between the higher energy states (which are lower-energy transitions, shown in purple, blue etc.) compared to states near the ground state (red, orange etc.) This anisotropy axis and parameter gives qualitative agreement with measured Eu-b spectra. (**d**) Simulated excitation energies for $D = +1.0$ GHz oriented the O-O bridge direction, assuming an in-plane magnetic field oriented 45° relative to the O-O direction. The first four excitation energies are seen to be nearly degenerate and thus not consistent with the measured Eu-b spectra. (**e**) Simulated ESR spectrum for hyperfine interaction strength $A = +700$ MHz, $D = +1.4$ GHz oriented along the surface normal similar to (c), with $B = 0.84$ T. These parameters are intended to qualitatively model the measured spectrum of ^{151}Eu shown in Figure S7d,e. Colors depict excitations between electron spin states, with all transitions for a given nuclear spin state colored the same. (**f**) Energy eigenvalues and (**g**) excitation energies for the model parameters used in (e) as a function of B. As B approaches zero, the spin states are no longer well-described as a product of electron and nuclear spin states, and instead the total angular momentum $F = I + J$ becomes a good quantum number for stationary states.

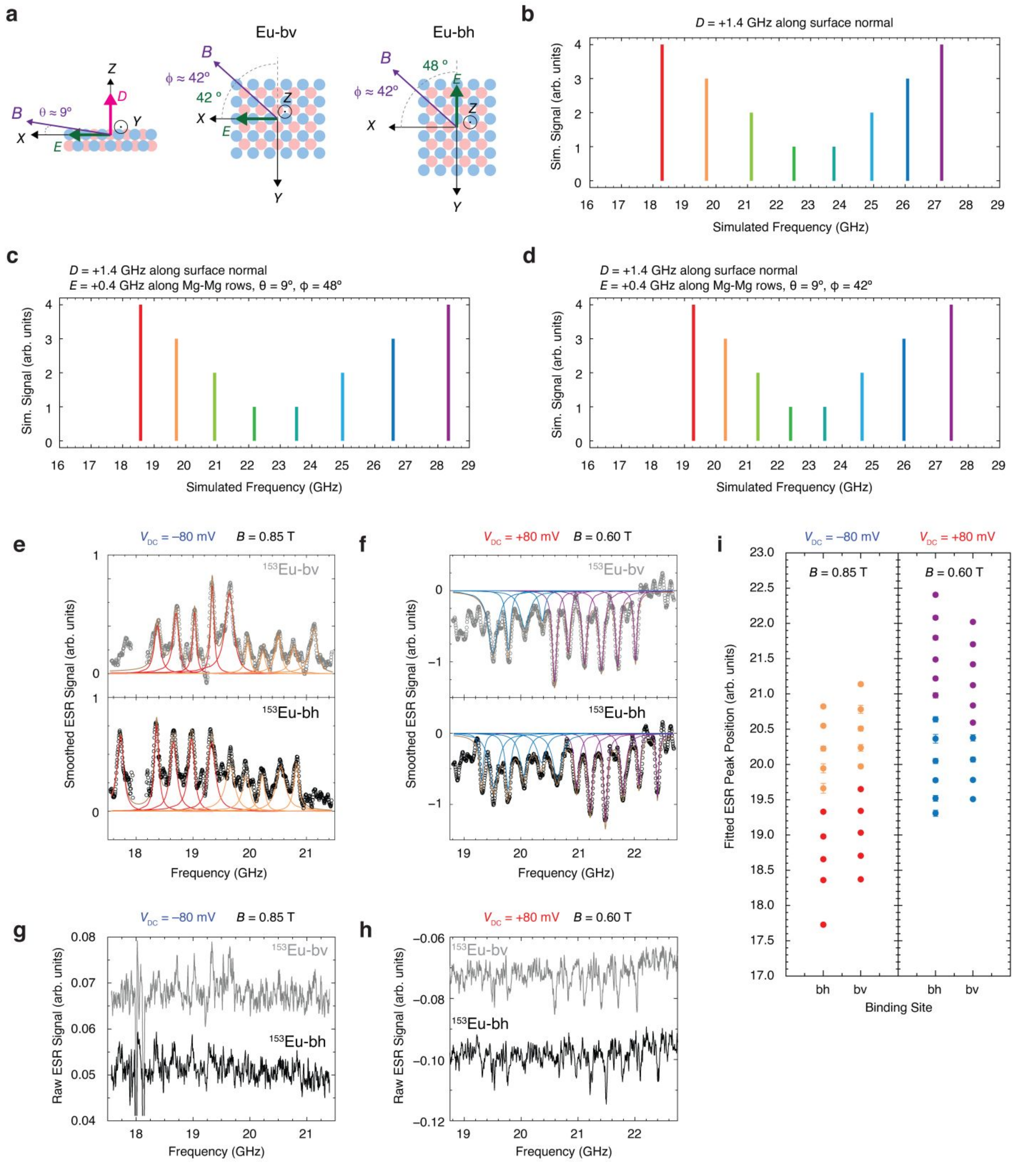

Figure S9. Effect of transverse crystal field *E* on model Hamiltonian for Eu-b.

(**a**) Schematic diagram depicting the orientation of the external magnetic field and Eu-b crystal field axes (*X, Y, Z*) with respect to the crystal surface. Left: side view showing the external field *B*, its out-of-plane angle θ and the axial anisotropy term *D*. Center and right: top-down views of Eu-bv and Eu-bh respectively, with the external magnetic field vector (purple arrow), in-plane angle φ, and transverse magnetic anisotropy term *E* (green arrow), which is oriented along the Mg-

Mg row direction. (**b**) Model Hamiltonian simulation of the excitation spectrum for Eu assuming D = +1.4 GHz oriented along the crystal Z axis, θ = 9° and E = 0, with the hyperfine coupling constant A is set to zero for simplicity. (**c**) Same as (b) except E = +0.4 GHz oriented along the Mg-Mg direction and φ = 48°. (**d**) Same as (c) except φ = 42°. These values for φ correspond approximately to the experimental external magnetic field orientations with respect to E for the two bridge directions. (**e**) Smoothed ESR spectrum of Eu-bv (top, gray) and Eu-bh (bottom, black) acquired at V_{DC} = −80 mV, tip-height setpoint I_{set} = 40 pA and V_{set} = +80 mV; V_{RF} = 30 mV, B = 0.85 T. (**f**) Smoothed ESR spectrum of Eu-bv and Eu-bh acquired at V_{DC} = −80 mV, I_{set} = 40 pA, V_{set} = +80 mV, V_{RF} = 30 mV, B = 0.60 T. Here B was chosen to reduce the frequency of peaks in trees 1 and 2 to the most transmitting RF window of our instrument transfer function, so that they appear in a similar range as trees 7 and 8 in (e). (**g**, **h**) Raw ESR spectra of (e) and (f), without smoothing, peak fits, or removal of artifact-dominated points. (**i**) Fitted peak positions for the data shown in (e) and (f). The redshift of excitations for Eu-bh in trees 7 and 8 (purple and blue points) and blueshift of excitations for Eu-bh in trees 1 and 2 (red and orange points) relative to comparable peaks for Eu-bv is predicted by the transverse magnetic anisotropy term E as shown in (c) and (d). In contrast, anisotropy in the Landé g-factor along the Mg-Mg and O-O bridge directions would cause the peaks of all trees for Eu-bh to redshift or blueshift compared to Eu-bv. In other words, the presence of transverse anisotropy E causes the two different Eu-b adsorption sites to appear to have different crystal field splitting when φ differs significantly from 45°. In contrast, g-factor anisotropy would cause the two different Eu-b adsorption sites to have the same crystal field splitting, and an overall frequency offset for all peaks caused by the different effective g-factors. The presence of an out-of-plane axial anisotropy D without a transverse anisotropy E would cause Eu-bv and Eu-bh to have identical spectra.

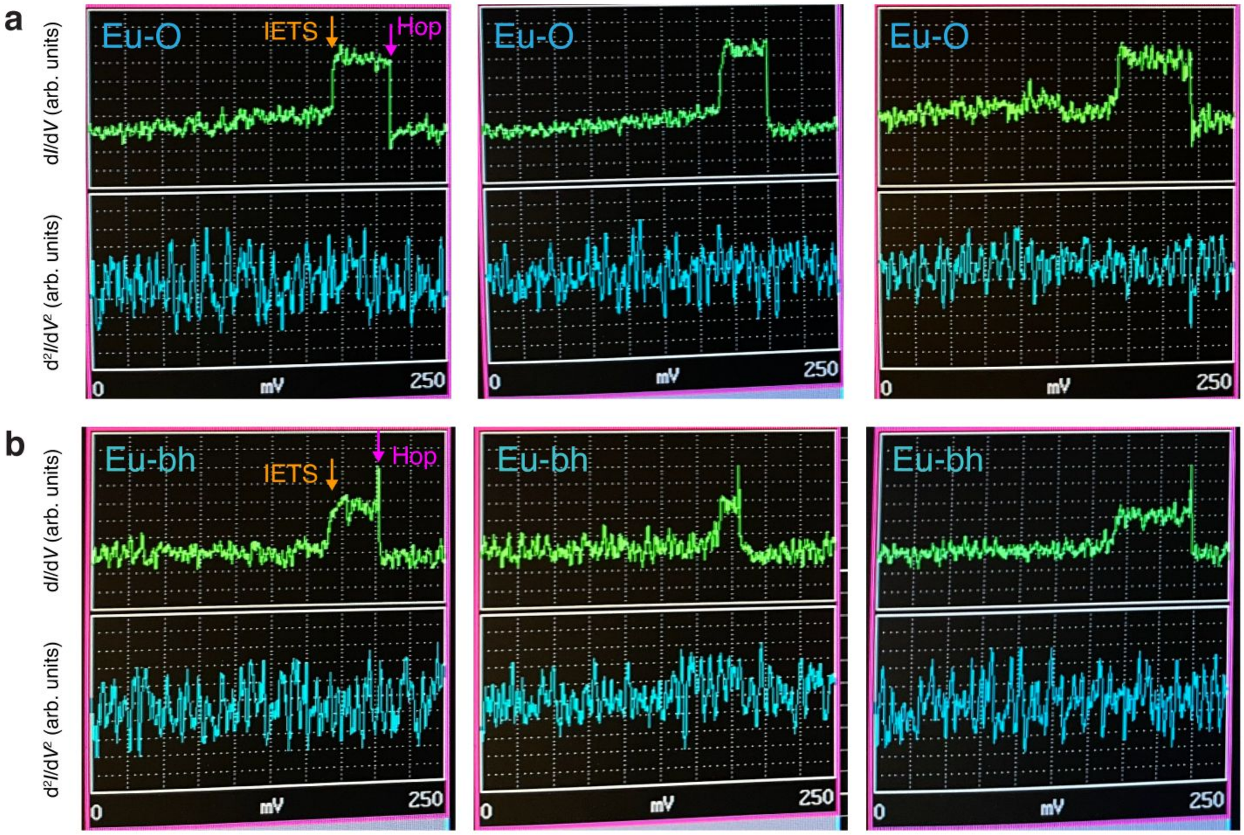

Figure S10. Tunneling spectra of Eu at oxygen and bridge sites.

(**a**, **b**) Three dI/dV traces each for Eu-O ((a), top row) and Eu-bh ((b), bottom row). Top (green) traces in each panel are dI/dV. Bottom (blue) traces are d^2I/dV^2, which are not reliably distinguishable from noise in these rapidly acquired spectra. The upward conductance step in dI/dV (~163 mV for Eu-b and ~165 mV for Eu-O) is the "6s-flip": the intra-atomic spin-flip excitation shown in main text Figure 1(e),(g). The downward conductance steps at ~180–220 mV result from the Eu atom hopping to the lower conductance Mg site because of the elevated bias voltage. The dI/dV traces shown here were acquired using a fast dI/dV acquisition mode of our instrument that enables rapid characterization of atoms, but which does not store the resulting data. Here we show photographs from the data-acquisition computer display. These spectra lead us to assign an open-shell ($6s^1$) configuration to Eu-O and to Eu-b, where the conductance steps visible here correspond to 6s-flip excitations, as for Sm-O and Sm-b. For Eu-O and Eu-b this transition reduces total spin S from 4 to 3 (term $^9S_4$ to $^7S_3$), as for the free Eu^+ ion excitation at 207 mV. Regrettably, this tip apex was lost before quantitative data could be recorded for Eu-O, and this particular tip was the only tip apex with which we could readily move Eu to the O-site and acquire tunneling spectra without the Eu hopping to an Mg site. We show this limited data to provide qualitative evidence that Eu-O is in the same open valence shell configuration as Eu-b, similar to Sm-O and Sm-b respectively. The Eu-O atom studied here was the same as that shown in the topograph in Figure S1d.

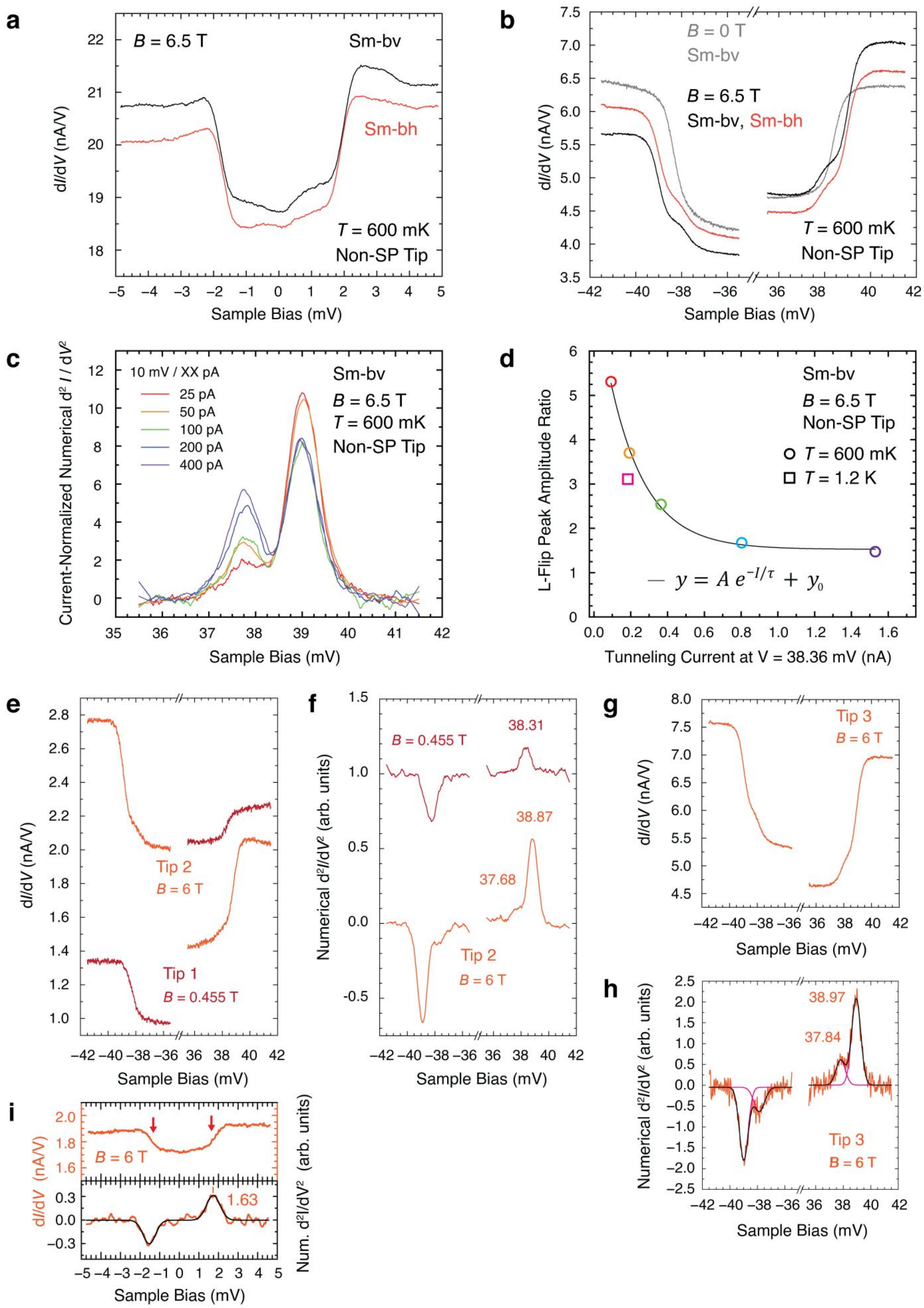
a
B = 6.5 T
Sm-bv
Sm-bh
T = 600 mK
Non-SP Tip
dI/dV (nA/V)
Sample Bias (mV)
b
B = 0 T
Sm-bv
B = 6.5 T
Sm-bv, Sm-bh
T = 600 mK
Non-SP Tip
dI/dV (nA/V)
Sample Bias (mV)
c
10 mV / XX pA
25 pA
50 pA
100 pA
200 pA
400 pA
Sm-bv
B = 6.5 T
T = 600 mK
Non-SP Tip
Current-Normalized Numerical d² I / dV²
Sample Bias (mV)
d
Sm-bv
B = 6.5 T
Non-SP Tip
T = 600 mK
T = 1.2 K
— y = A e^{-I/τ} + y₀
L-Flip Peak Amplitude Ratio
Tunneling Current at V = 38.36 mV (nA)
e
Tip 2
B = 6 T
Tip 1
B = 0.455 T
dI/dV (nA/V)
Sample Bias (mV)
f
B = 0.455 T
38.31
38.87
37.68
Tip 2
B = 6 T
Numerical d²I/dV² (arb. units)
Sample Bias (mV)
g
Tip 3
B = 6 T
dI/dV (nA/V)
Sample Bias (mV)
h
38.97
37.84
Tip 3
B = 6 T
Numerical d²I/dV² (arb. units)
Sample Bias (mV)
i
B = 6 T
1.63
dI/dV (nA/V)
Num. d²I/dV² (arb. units)
Sample Bias (mV)

Figure S11. Tunneling spectra for Sm at bridge sites.

(**a**) Low-bias tunneling spectra (dI/dV) of bridge-site Sm atoms (Sm-bh and Sm-bv) show Zeeman splitting of the ground state at B = 6.5 T. The numerical derivatives of these spectra (d^2I/dV^2) are shown in main text Figure 4c. Anisotropy in the g-tensor, combined with the effect of the external magnetic field being more oriented along the O-O bridge direction for Sm-bh and more oriented along the Mg-Mg bridge direction for Sm-bv, results in the different energies for the two Sm-b orientations seen here and in Figure 4c. A Gaussian fit to each peak in Figure 4c, averaged over positive and negative bias, gives energies 1.786 ± 0.004 mV for Sm-bh and 1.893 ± 0.004 mV for Sm-bv. These energies correspond to g = 4.747 for Sm-bh and g = 5.031 for Sm-bv. Tip-height setpoint I_{set} = 200 pA at V_{set} = 10 mV, V_{mod} = 0.2 mV rms. (**b**) Tunneling spectra of Sm-b shows magnetic-field splitting of the ~38-mV peak. The numerical derivatives of these spectra (d^2I/dV^2) are shown in Figure 4d. Splitting is symmetrical in energy but shows different peak heights. The effect of the bridge orientation (Sm-bv versus Sm-bh) is negligible. Tip-height setpoint I_{set} = 50 pA at V_{set} = 10 mV, V_{mod} = 0.2 mV rms. (**c**) Tip-height dependence of tunneling spectra of Sm-bv with tip height setpoint of 10 mV at current as labeled. Each spectrum is normalized by the tunneling current measured at 38.3 mV, which is the bias onset of the L-S-tilt excitation. (**d**) Ratio of peak-heights of the split ~38 mV excitation from fits to 0.6 K data (open circles, spectra shown (**c**)) and at 1.2 K (open square, spectra not shown). (**e**) Tunneling spectra (dI/dV) for Sm-b at B = 0.455 T (dark red curves) and B = 6 T (orange curves). The data was acquired with two different tip apexes as labeled, which both differed from the apex used for the spectra shown in (b) and Figure 4d. Both sets of spectra used tip-height setpoint I_{set} = 20 pA at V_{set} = 10 mV, V_{mod} = 0.2 mV rms. (**f**) Corresponding numerical derivatives d^2I/dV^2 of spectra in (e). The derivative used Savitzky-Golay smoothing in a 20-point window. (**g**) Additional tunneling spectra for Sm-b acquired at B = 6 T with a different tip apex ("Tip 3") and (**h**) corresponding numerical derivative smoothed with a 20-point window. Data shown in (g) and (h) were acquired at setpoint I_{set} = 50 pA at V_{set} = 10 mV, V_{mod} = 0.2 mV rms. (**i**) Additional low-bias tunneling spectra (dI/dV, top) and corresponding numerical derivative (d^2I/dV^2, bottom) of Sm-bh showing the Zeeman splitting of the ground state doublet, acquired with a different tip apex than was used in (a) and Figure 4c. Tip-height setpoint 200 pA at 10 mV, V_{mod} = 0.2 mV rms.

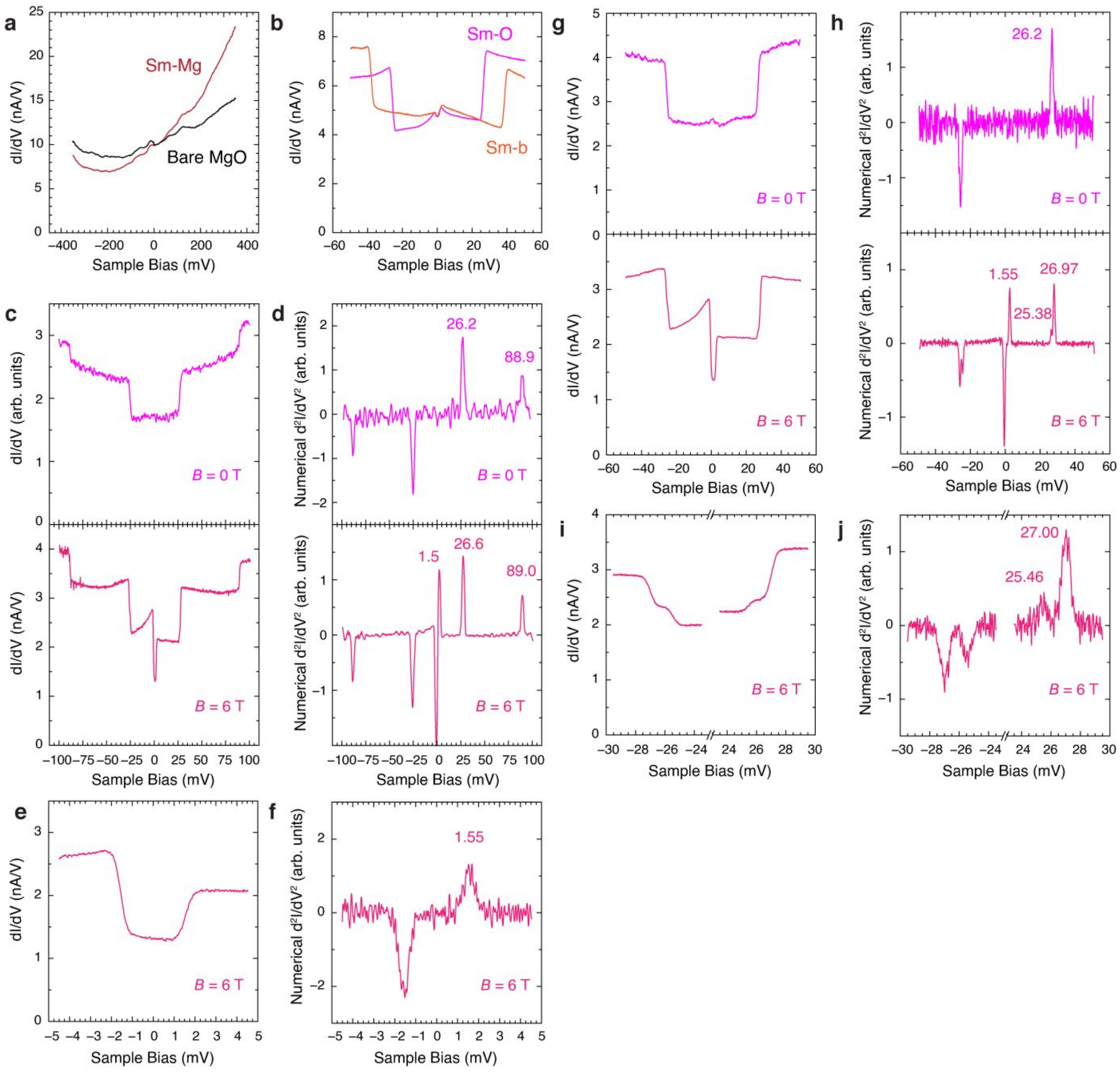

Figure S12. Tunneling spectra of Sm at Mg and oxygen sites.

(**a**) Tunneling spectra (dI/dV) acquired with the tip positioned over Sm-Mg (dark red) and the bare MgO substrate (black) using the same STM tip apex. Tip-height setpoint I_{set} = 100 pA at V_{set} = 10 mV. (**b**) Tunneling spectra of Sm-b (orange) and Sm-O (pink). Setpoint I_{set} = 10 pA at V_{set} = 50 mV, V_{mod} = 1 mV rms, B = 0.85 T. The same tip apex was used for both traces, but the apex used in (b) differs from that used in (a). (**c**) Tunneling spectra of Sm-O acquired at larger tip-surface distance and larger bias voltage range, showing the conductance step at ~89 mV. Acquired at B = 0 T (top) with a non-spin-polarized tip and B = 6 T (bottom) with a spin-polarized tip. Top spectrum: I_{set} = 5 pA at V_{set} = 20 mV, V_{mod} = 1 mV rms. Bottom spectrum: I_{set} = 5 pA at V_{set} = 20 mV. (**d**) Numerically computed d^2I/dV^2 spectra of corresponding traces in (c). (**e**) Low-voltage tunneling spectrum measuring the Zeeman splitting of Sm-O at B = 6 T, acquired with the same spin-polarized tip as bottom panels of (c) and (d). Setpoint I_{set} = 20 pA at V_{set} = 10 mV, V_{mod} = 0.2 mV rms at T = 0.6 K. (**f**) Numerically computed d^2I/dV^2 spectrum of (e). (**g**) Zoom-in bias

range at $B = 0$ T (top) with a non-spin polarized tip and $B = 6$ T (bottom) with a spin-polarized tip. Top: $I_{set} = 25$ pA at $V_{set} = 10$ mV, $V_{mod} = 1$ mV rms at $T = 1.2$ K. Bottom: $I_{set} = 20$ pA at $V_{set} = 10$ mV, $V_{mod} = 0.3$ mV rms at $T = 1.2$ K. (**h**) Numerically computed d^2I/dV^2 spectra of corresponding traces in (g). (**i**) Tunneling spectra showing the splitting of the ~26 mV conductance step at $B = 6$ T. $I_{set} = 20$ pA at $V_{set} = 10$ mV, $V_{mod} = 0.2$ mV rms at $T = 0.6$ K. (**j**) Numerically computed d^2I/dV^2 spectra of corresponding traces in (i).

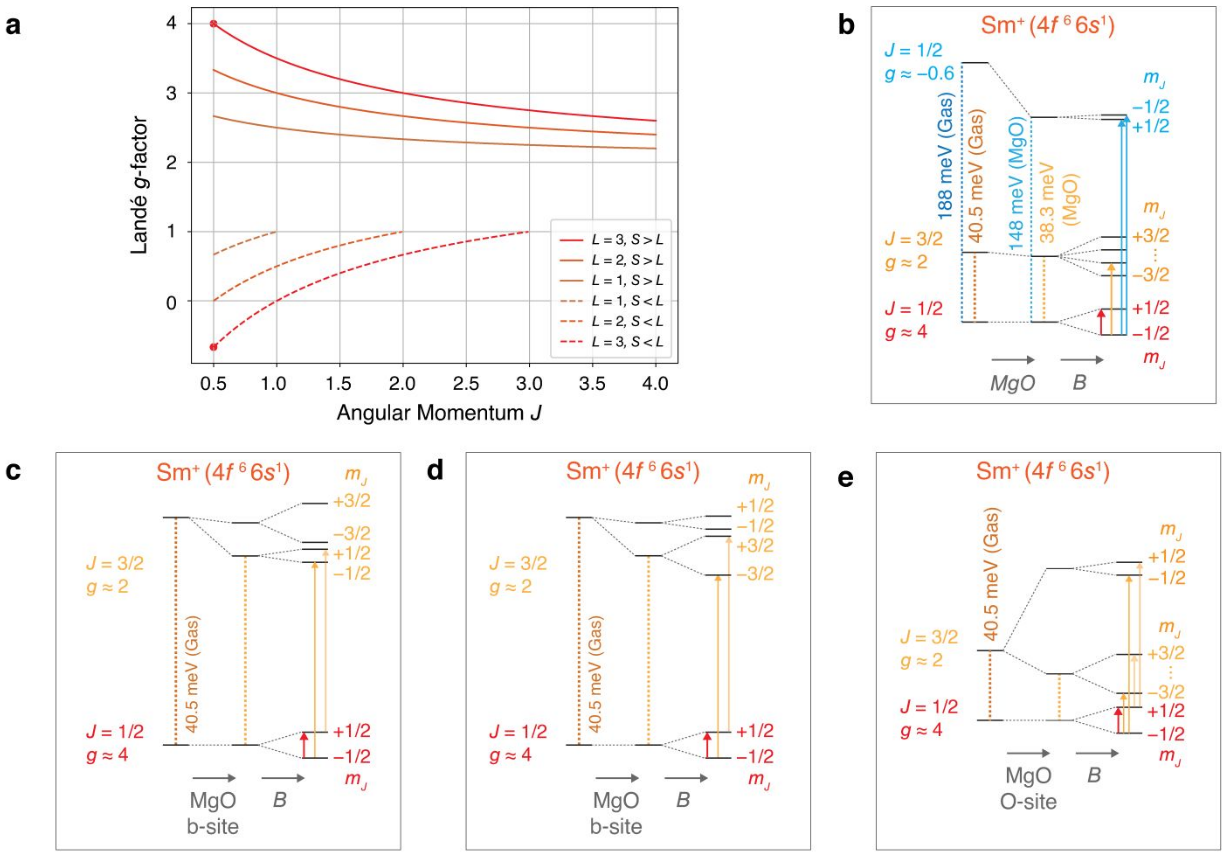

Figure S13. Proposed excitation schemes and calculated Landé *g*-factors for Sm^+

(**a**) Calculated Landé *g*-factors for various L as a function of J, including the case of $L = 3$ found in gas-phase Sm^+ (red). Red points correspond to the $L = 3$, $J = 1/2$ cases found in free Sm^+ ground and excited multiplets. Hund's third rule is assumed, so $J = |S - L|$. Lines show J varied continuously for illustration. Large *g*-factors occur when J is non-zero but minimized. (See Supplementary Note 7) (**b**) Energy level scheme for gas-phase Sm^+, and for Sm at a bridge or oxygen site on MgO (Sm-b or Sm-O), with arrows showing IETS transitions, reproduced from main text Figure 4e. Crystal field splitting is assumed to be near zero. (**c**) Alternative excitation scheme in which the $J = 3/2$ excited multiplet of Sm is split into two m_J doublets by the crystal field when adsorbed on the b-site, with the $m_J = \pm 1/2$ doublet lower in energy than the $m_J = 3/2$ doublet. (**d**) Alternative level scheme similar to (c) but with the $m_J = \pm 3/2$ doublet lower in energy. (**e**) Alternative excitation scheme for Sm adsorbed on the O-site in which the crystal field splitting is larger. (See Supplementary Note 5 for discussion of (b–d) and Supplementary Note 6 for (e)).

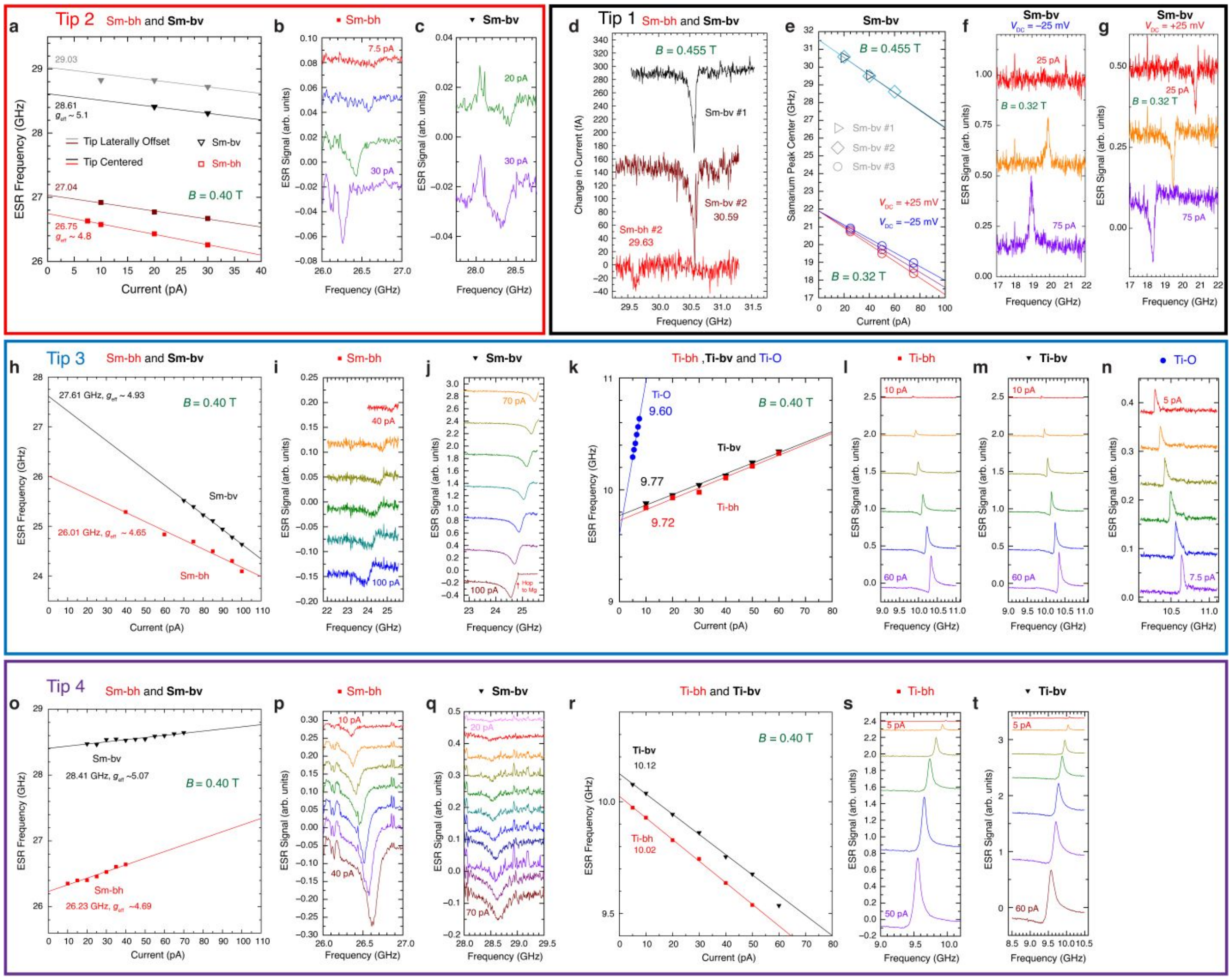

Figure S14. Anisotropic *g*-factor determination from ESR spectra of Sm-b.

(**a**) Tip-sample distance dependent ESR peak positions for Sm-bh (bridge-site, horizontal O-O orientation, squares) and Sm-bv (bridge-site, vertical orientation, triangles) acquired with the tip positioned laterally above the center of the atom (grey and brown points) or laterally offset ~0.15 nm (black and red points). All spectra acquired with V_{DC} = +25 mV, B = 0.40 T, and tip-height varied by varying the setpoint current I_{set} as shown, at V_{set} = 25 mV. (**b**) Raw data for Sm-bh and (**c**) raw data for Sm-bv, which yielded the red and black data points in (a) for the tip centered over the atom. All data in (a–c) was acquired with the same tip apex "Tip 2" and the same individual isolated Sm atom, which was repositioned between the bh and bv sites via atom manipulation. (**d**) ESR spectra comparing Sm-bh and Sm-bv with a different tip apex, "Tip 1". (**e**) Fitted ESR peak positions as a function of setpoint current for several Sm-bv atoms at different magnetic fields B. The data shown for B = 0.32 T was acquired for both positive and negative bias polarity at the same tip-surface distance (open feedback loop after setting the tip height), demonstrating a subtle bias-voltage dependence on ESR frequency, an effect that has been noted recently for transition metal adatoms[20]. The purple points indicate the average of red and blue data points. Data shown for B = 0.455 T was acquired at V_{DC} = +25 mV for multiple individual Sm atoms as indicated inset. (**f**) Raw data for spectra shown at B = 0.32 T for negative bias and (**g**)

positive bias. The spectra in (f) and (g) have been normalized by the setpoint current. (**h**) Additional ESR spectra comparing Sm-bh and Sm-bv with tip apex "Tip 3", along with raw data for (**i**) Sm-bh and (**j**) Sm-bv, similar to (a–c). All Sm spectra were acquired with bias V_{DC} = +25 mV with V_{set} = +25 mV and setpoint current as shown. (**k**) Fitted ESR peak positions using the same tip apex "Tip 3" to acquire data on Ti-bh, Ti-bv and Ti-O atoms. (**l**–**n**) Raw data for Ti-bh, Ti-bv and Ti-O, respectively, at different I_{set} as labeled. Spectra vertically offset for clarity. All Ti spectra were acquired with bias V_{DC} = +50 mV, V_{set} = +50 mV and setpoint current as shown. (**o**–**t**) Additional ESR spectra of Sm-bh, Sm-bv, Ti-bh, and Ti-bv acquired with a fourth tip apex, "Tip 4." All data for Sm and Ti were acquired under similar conditions as for "Tip 3" in (h–n). All Sm spectra: V_{RF} = 10 mV; all Ti spectra: V_{RF} = 20 mV. Combining the extrapolated zero-current intercepts for tips 2–4 shown here gives an average effective *g*-factor of 5.04 ± 0.08 for Sm-bv and 4.71 ± 0.05 for Sm-bh.

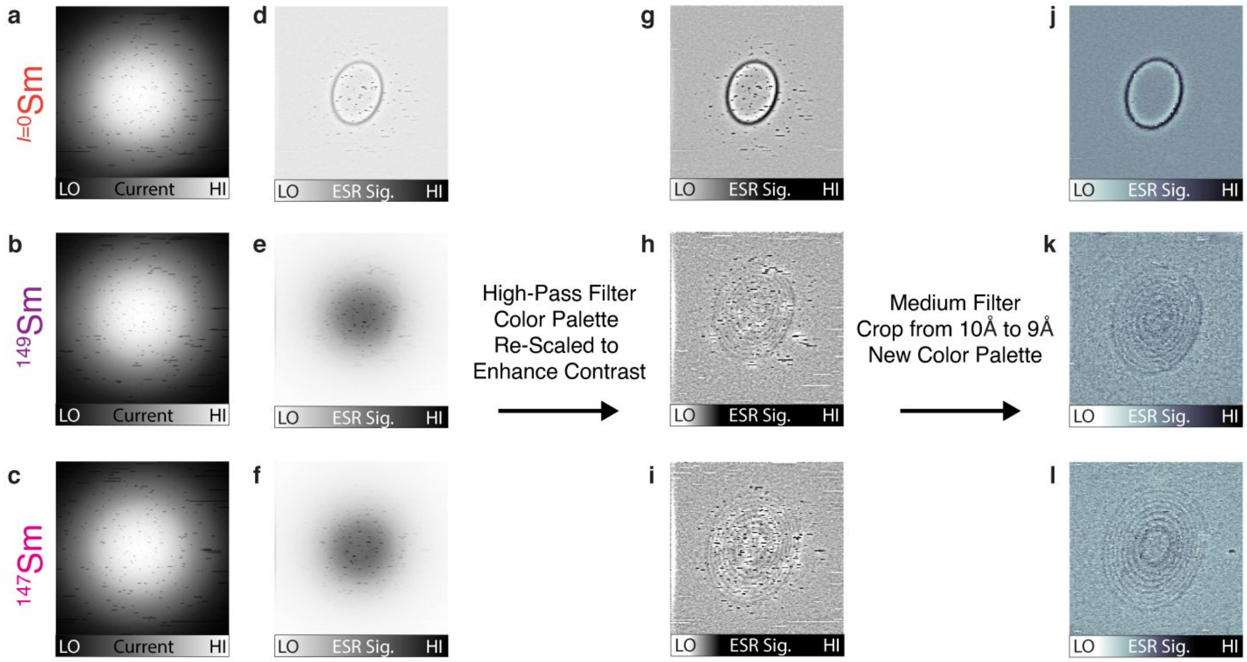

Figure S15. Post-processing of Sm magnetic resonance image (MRI) data.

(**a**–**c**) Tunneling current at constant tip height for $^{I=0}$Sm, ^{149}Sm and ^{147}Sm (respectively). The dark dots and lines ("popcorn" noise) resulted from a magnetically bi-stable STM tip and were not an intrinsic property of the Sm atoms. (**d**–**f**) Raw magnetic resonance images (MRI) for each Sm isotope acquired simultaneously with the current maps shown in (a–c). The ESR signal for $^{I=0}$Sm results in a single, clearly visible dark resonant slice that appears as an oval shaped ring. The smaller ESR signal for each resonant slice of the I = 7/2 nuclear isotopes (e) and (f) results in a series of concentric dark resonant slices which are not immediately obvious against the rectification background (diffuse dark region centered on the atom) or the popcorn noise. (**g**–**i**) High-pass filtered data from (d–f) rescaled non-linearly to emphasize the contrast arising from the ESR signal. Here the high-pass filtering had the effect of removing the rectification background which varied slowly with lateral tip position while preserving the ESR signals which varied rapidly with tip position. (**j**–**l**) Cropped, median filtered, high-pass filtered images plotted with an alternative color palette that was similarly adjusted to emphasize contrast (non-linear). Here median filtering served to remove the popcorn noise arising from switching of the tip magnetization during image acquisition. Each image was cropped from 1 nm to 0.9 nm to remove the imaging artifact at the start of each horizontal scan line (bright edge seen on the left of each image in (g–i)).
